# Megapixel snapshot hyperspectral imaging with high resolution Fabry–Pérot color filter array

**DOI:** 10.1093/nsr/nwag526

**Published:** 2026-08-22

**Authors:** Yu Chen, Jiaming Huang, Zixian Hu, Rong Rong, Qichang Ma, Peiqi Zeng, Jiafei Chen, Hoilam Tam, Junhong Deng, Kingfai Li, Kokwai Cheah, Guixin Li

**Affiliations:** Department of Materials Science and Engineering, Southern University of Science and Technology, Shenzhen 518055, China; Department of Physics, Hong Kong Baptist University, Hong Kong 999077, China; Department of Materials Science and Engineering, Southern University of Science and Technology, Shenzhen 518055, China; Department of Materials Science and Engineering, Southern University of Science and Technology, Shenzhen 518055, China; School of Integrated Circuits, Harbin Institute of Technology, Shenzhen 518055, China; Department of Materials Science and Engineering, Southern University of Science and Technology, Shenzhen 518055, China; Department of Materials Science and Engineering, Southern University of Science and Technology, Shenzhen 518055, China; School of Integrated Circuits, Harbin Institute of Technology, Shenzhen 518055, China; Department of Materials Science and Engineering, Southern University of Science and Technology, Shenzhen 518055, China; Department of Materials Science and Engineering, Southern University of Science and Technology, Shenzhen 518055, China; Department of Materials Science and Engineering, Southern University of Science and Technology, Shenzhen 518055, China; Department of Physics, Hong Kong Baptist University, Hong Kong 999077, China; Department of Materials Science and Engineering, Southern University of Science and Technology, Shenzhen 518055, China

**Keywords:** hyperspectral imaging, Fabry–Pérot cavity, color filter array, grayscale lithography

## Abstract

In the field of snapshot hyperspectral imaging, a long-pursuit objective is to develop advanced spectral information encoding elements with both high spatial resolution and large sensor size. Among various strategies, Fabry–Pérot (F–P) cavity color filter, which has both high transmittance and narrow-color filtering function, represents a promising platform. However, it is extremely challenging to make F–P color filters by using multi-layer deposition method. Here, we demonstrate that a commercial-sensor-compatible F–P cavity color filter array can be fabricated through the single-step grayscale electron beam lithography. In the proof-of-concept experiment, the silver–dielectric–silver F–P color filter array, which has spatial resolution of 5.2 µm × 5.2 µm and pixel number of 1.26 million, is successfully used to demonstrate the hyperspectral imaging function. As the diameter of the electron beam is at the level of tens of nanometers, the pixel size of the F–P cavity can be down to 3.45 µm × 3.45 µm and even further. The proposed method may have great potential in high-resolution snapshot hyperspectral imaging and related applications in optical inspection, remote sensing, biomedical imaging and so on.

## Teaser

A megapixel Fabry–Pérot color filter array fabricated with grayscale electron beam lithography allows for high-resolution snapshot hyperspectral imaging.

## INTRODUCTION

Hyperspectral imaging is a powerful technology for measuring and processing both the spatial and spectral information of an image. This technology represents a noninvasive solution for identifying and analyzing objects and materials, therefore it can be widely used for remote sensing in agriculture, environmental monitoring, optical inspection of industrial products and biomedical specimens. In the past years, the spatial- and spectral-scanning methods have been widely developed to achieve full-frame hyperspectral imaging. However, scanning-based methods are usually time-consuming. To solve this constraint, the digital micromirror device and spectrometer are jointly utilized to realize a scanning-free instrument for compressive hyperspectral imaging [1]. It has been shown that snapshot hyperspectral imaging camera holds the potential to comprise high spatial resolution, high spectral resolution and imaging speed. In conventional snapshot hyperspectral imaging, the optical systems are usually based on the dispersive optical element [2,3], diffractive optical element [4–9] and nonlinear crystals [10], which are bulky and thus difficult to be used for portable applications. Therefore, one of the key targets in this area is to develop complementary metal–oxide–semiconductor (CMOS) compatible spectral information encoding elements, such as dye-doped photoresist [11] and color ink [12]. Among various color filter designs, two strategies are usually used to balance the trade-off between high spatial resolution and high spectral resolution. The first strategy is based on the electrical tuning devices, such as single heterojunction [13–15], liquid crystals [16–18], phase change materials [19,20], micro-electro-mechanical systems (MEMS) integrated moiré photonic crystals [21], electro-optic device [22] and quantum dot [23]. Similarly, the color filter array based on humidity-sensitive materials has also been explored [24]. In these devices, the multi-step spectral information can be temporally modulated in an individual color filter without sacrificing the additional spatial channels. Nowadays, these devices suffer from the limited pixel number due to the complexity of fabrication. The second strategy is to reduce the pixel size of each color filter and maintain the large sensor size. To achieve this goal, photonic crystal [25] and metasurface [26,27] have been utilized to engineer the spectral responses of the color filters. In the reported hyperspectral imaging cameras, the pixel size of the photonic crystal and metasurface color filters is in the range of 5.8 µm × 5.8 µm to 32 µm × 32 µm [25,27–29]. In addition, 16 kinds of organic dyes and nano-metal oxides doped color filters with pixel size of 10 µm × 10 µm can be fabricated on a single hyperspectral sensor by using the multi-step photolithography processes [30]. However, increasing the number of spectral channels with this technique suggests that more kinds of dyes and lithography cycles need to be used. Last but not least is the well-known Fabry–Pérot (F–P) cavity color filters. By using a series of shadow masks with different randomness, multi-step vacuum deposition and etching processes, the F–P cavity color filters consisting of distributed Bragg reflectors and a cavity layer are successfully fabricated [31]. The minimum pixel size of the multilayer dielectric F–P color filter is 5.5 µm × 5.5 µm. However, further shrink of the pixel size of the F–P cavity or increasing the number of spectral channels may introduce serious overlay errors between the neighboring pixels. From the above analysis, we find that the fabrication of dye-type and F–P cavity color filters faces great challenges in terms of multiple overlay processes, pixel size scalability and time consumption. Therefore, it is highly desirable to develop advanced spectral information encoding technique for snapshot hyperspectral imaging.

In this work, we design and fabricate the high-resolution F–P cavity color filter arrays with random spatial distribution by using grayscale electron beam lithography technique. The F–P cavity is composed of a thin layer of electron resist (Poly(methyl methacrylate), PMMA) with spatially variant thickness and two semi-transparent silver thin films as the cavity mirrors. A single-step grayscale electron beam lithography is used to define the spectral responses of all the color filters. This is realized by controlling the electron exposure dose to the PMMA resist which consequently changes the spacer thickness of the F–P cavities. In the visible regime, the total thickness of the F–P cavity color filters is less than 250 nm. In previous works [32–34], the F–P cavity color filter arrays have been fabricated by using similar method but have not been used for high-resolution hyperspectral imaging measurement. Here, we develop a series of megapixel F–P cavity color filter arrays which have randomly distributed spectral encoding channels with spatial resolution of 6.9 µm × 6.9 µm and 5.2 µm × 5.2 µm, respectively. By using the compressed sensing and image reconstruction algorithm, the megapixel F–P cavity color filter arrays are successfully utilized for snapshot hyperspectral imaging. To further demonstrate the scalability of the grayscale electron beam lithography, the F–P color filter array with a pixel size of 3.45 µm × 3.45 µm is experimentally realized and exhibits excellent spectrometer function. In the visible regime, the peak transmission efficiency of the fabricated F–P cavity color filters is above 60%. While featuring both high spatial resolution and high transmittance, the proposed Ag/PMMA/Ag F–P cavity color filter array and the corresponding fabrication process in this work are suitable for large-scale mass production, which, in our view, represents an advanced CMOS-compatible strategy for snapshot hyperspectral imaging.

## RESULTS AND DISCUSSION

### Hyperspectral imaging with the randomly distributed F–P cavity color filters

The working principle of the snapshot hyperspectral imaging with the randomly distributed F–P cavity color filters is shown in Fig. 1a. When light from the object passes through the F–P cavity color filters, it is encoded by the measurement matrix and finally captured by the monochrome camera to form a compressed image. Then, the compressed sensing method is used to reconstruct both the spatial and spectral information of the object under the spatial sparsity approximation [31,35]. It is known that the spatial and spectral randomness are important for reducing the reconstruction error. Therefore, we choose the same strategy when designing the randomly distributed Ag/PMMA/Ag F–P cavity color filters (Fig. 1b). As shown in Fig. 1c, in the visible regime, the transmission peak of the F–P cavities can be continuously tuned by varying the thickness of the PMMA spacer layer. By sequentially illuminating the random color filter array using laser with different wavelengths, we are able to obtain a measurement matrix which contains the spectral responses of all the spatially variant encoding channels (Fig. 1d). Based on the Two-Step Iterative Shrinkage/Thresholding (TwIST) algorithm [36], the hyperspectral images (Fig. 1e) are reconstructed from the recorded monochrome image (Note S1).

**Figure 1.**
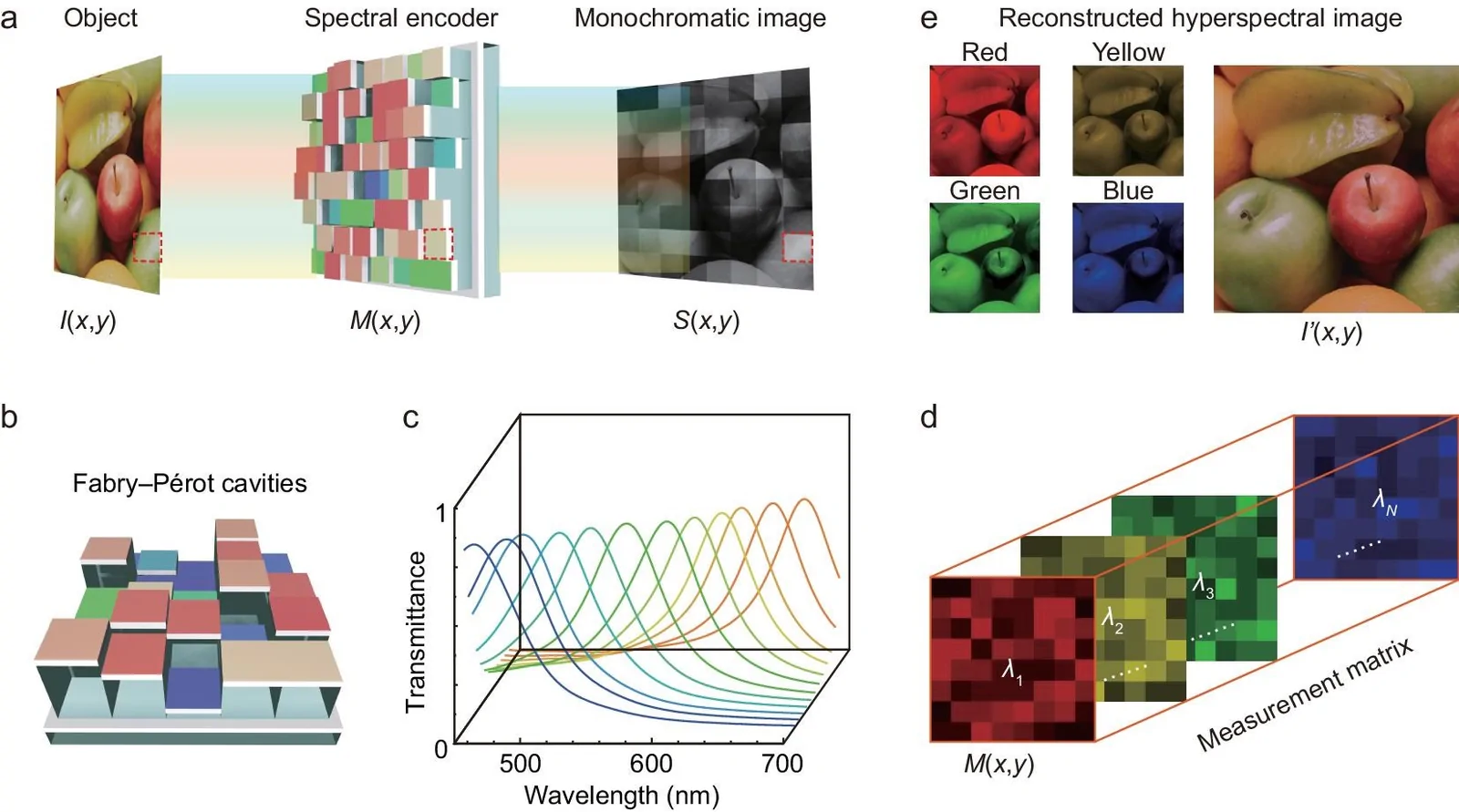
Working principle of the hyperspectral imaging system based on grayscale electron beam lithography enabled F–P cavity color filter array. (a) Schematic illustration of the hyperspectral imaging process with the F–P cavity color filter array (spectral encoder). After passing through the spectral encoder, the color image of an object is recorded by a monochrome complementary metal–oxide–semiconductor (CMOS) sensor. (b) Schematic diagram of the randomly distributed F–P cavity color filters which are fabricated on a silica substrate. Two semi-transparent silver thin films (∼30-nm-thick) act as the mirrors and the electron resist (PMMA) layer with spatially variant thickness is used as the cavity spacer. On top of each cavity, a 20-nm-thick silicon dioxide layer is deposited to reduce the oxidation effect of silver. (c) Typical transmission spectra of the F–P cavities in the visible regime. As the thickness of spacer increases, the peak of the transmission spectrum shifts to longer wavelengths. (d) Illustration of the measurement matrix for hyperspectral imaging. The measurement matrix represents the spatial and spectral information of the randomly distributed F–P cavity color filters. (e) Reconstructed hyperspectral images. The recorded monochromatic image in (a) and the measurement matrix (d) are used to reconstruct the hyperspectral images by using the iterative algorithm.

### Optical properties of the silver/PMMA/silver F–P cavities

The F–P cavity color filter array is fabricated on the fused silica substrate and encapsulated by a ∼20-nm-thick silicon dioxide (SiO_2_) thin film to reduce the oxidation effect of silver. Firstly, the transmission spectra of the Ag/PMMA/Ag F–P cavities as a function of the PMMA thickness are numerically calculated by using the Lumerical FDTD software. To balance the transmittance and the bandwidth of the F–P cavity mode, the thickness of silver mirrors is chosen to be 25 nm in the calculation model. From the results in Fig. 2a, b, the thickness of the Ag/PMMA/Ag layers is 25 nm/80 nm–180 nm/25 nm, and the transmission peak shifts to longer wavelength as the PMMA thickness increases. The refractive indices of PMMA and silver are from the measured results by using the spectroscopic ellipsometer and the previous literature [37], respectively (Note S2).

**Figure 2.**
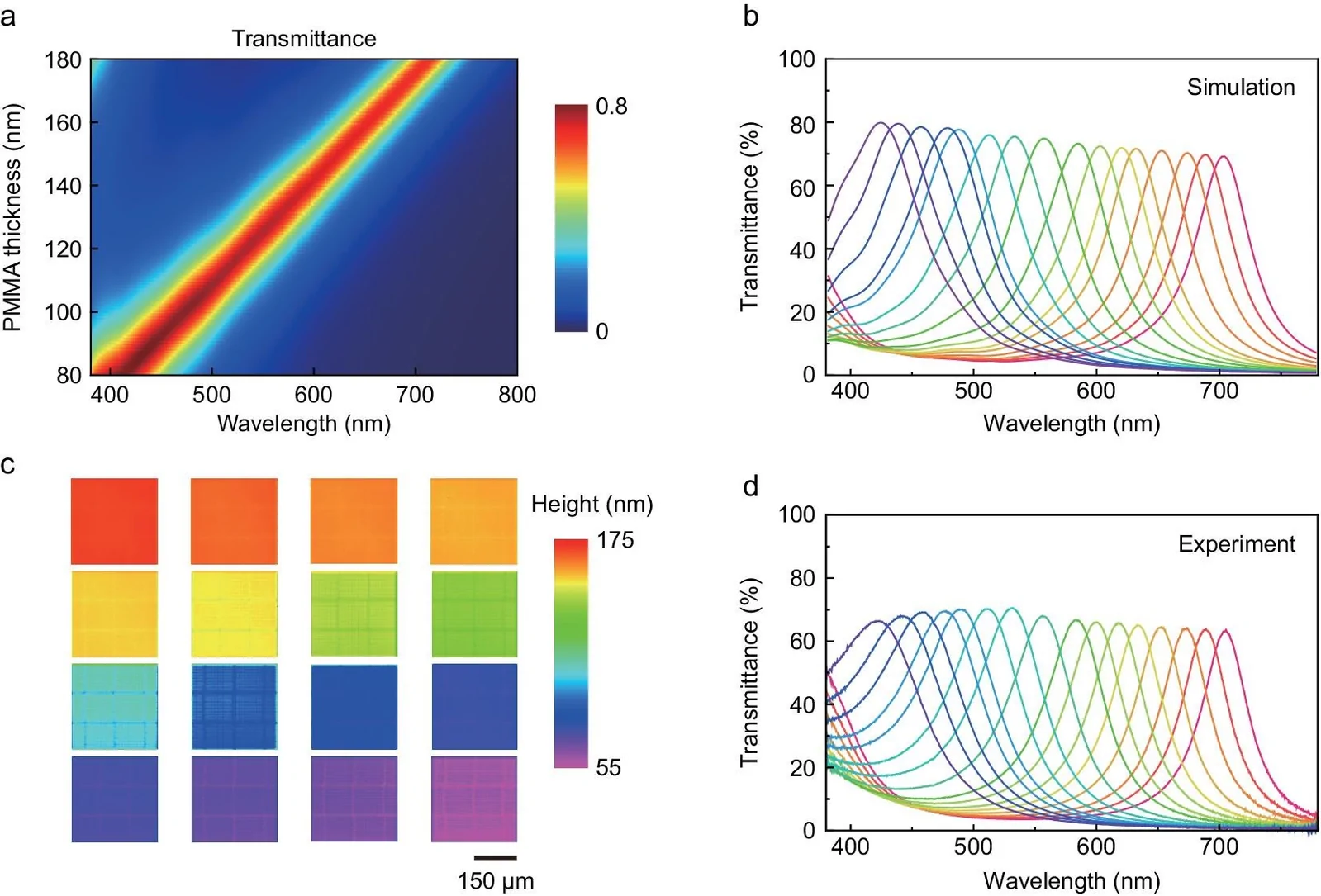
Optical properties of the F–P cavity color filters fabricated with grayscale electron beam lithography. (a) Simulated transmission spectra of the Ag/PMMA/Ag (25 nm/80 nm–180 nm/25 nm) F–P cavity color filters. All the cavities are encapsulated by a 20-nm-thick SiO_2_ layer. Red shift of the peak wavelength of the transmission spectra is observed as the PMMA thickness increases. (b) By judiciously choosing the PMMA thickness, the transmission spectrum of the F–P cavity exhibits one peak in the visible regime. The peak transmission efficiency of all the cavities is between 70% and 80%. (c) Height profile of the fabricated Ag/PMMA/Ag (∼30 nm/55.6 nm–174.6 nm/30 nm) F–P cavities measured by using white light interferometer. (d) Measured transmission spectra of the F–P cavities. All the cavities are encapsulated by a ∼20-nm-thick SiO_2_ layer. The measured transmission efficiency is between 65% and 70%, which is very close to the simulated results in (b).

To experimentally investigate the optical performance of the F–P cavities, a series of color palettes with area size of 300 µm × 300 µm is fabricated by using grayscale electron beam lithography technique. Firstly, the bottom silver layer with thickness of ∼30 nm is deposited on the fused silica substrate. Then, the PMMA resist with thickness of ∼207 nm is spin-coated onto the silver layer and patterned by using the electron beam lithography system. By controlling the exposure dose (∼75–246 µC/cm^2^), the thickness of PMMA layer can be controlled at the resolution of ∼3.0 nm/step. The beam current is set as 10 nA to balance the high exposure speed (∼4800 µm^2^/s) and small beam size (<30 nm). After depositing the top silver mirror with thickness of ∼30 nm and the 20-nm-thick silicon dioxide encapsulation layer, the color palettes with various cavity thickness are obtained. The height profiles of the F–P cavities are confirmed by the white light interferometry measurement results, as shown in Fig. 2c (Note S3). The measured transmission spectra of the color palettes are summarized in Fig. 2d (Note S4). It is shown that when the PMMA spacer thickness is varied from 55.6 to 174.6 nm, the peak wavelength of the F–P cavity is shifted from 422 to 705 nm. The peak transmission efficiency of all the cavities is above 60%, which is close to that in the calculated results (Fig. 2b). The full width at half maximum (FWHM) of the transmission peaks for the red (634 nm), green (531 nm) and blue (459 nm) F–P cavities are ∼54, 72 and 116 nm, respectively, which are narrower than that of commercial color filters [38]. By combining the grayscale electron beam lithography technique with the design of the Ag/PMMA/Ag F–P cavities, we are able to build an excellent library for tuning the spectral response of the color filters, which can be used for hyperspectral imaging in the visible regime.

### Hyperspectral imaging with the 6.9 µm × 6.9 µm F–P cavity color filters

To verify the hyperspectral imaging performance with the F–P cavity color filters, a randomly distributed spectral encoder, which contains cavities with 16 kinds of spacer thicknesses, is designed. The transmission peaks are homogeneously distributed in the visible regime. The sparsity of the object and the randomness of the color filters are verified by the simulated imaging and reconstruction process to make sure of the high-accuracy hyperspectral imaging reconstruction (Fig. S6). The hyperspectral image dataset is from the Columbia Imaging and Vision Laboratory (CAVE) at Columbia University, and the measurement matrix of the randomly distributed color filters is from the calculated one in Fig. 2b. The averaged root mean square error (RMSE) between the reconstructed spectra and the ground truth is ∼0.04 (Note S5). We then use grayscale electron beam lithography to fabricate the random color filter array with active pixel number of 532 × 712. The pixel size of each color filter is 6.9 µm × 6.9 µm which is equivalent to 2 × 2 pixels of the monochrome CMOS sensor (CS165MU, Thorlabs). In the hyperspectral imaging experiment, the intensity values of the 2 × 2 pixels on the CMOS sensor are averaged to yield an intensity signal. Therefore, the spatial resolution is mainly determined by the pixel size of the color filter array. In the following demonstration, we also fabricate the random color filter array with pixel size of 3.45 µm × 3.45 µm, where each color filter only covers one pixel on the CMOS sensor, providing higher active pixel number of 1060 × 1420. Therefore, the reduction of the color filter size could improve the spatial resolution of the whole hyperspectral imaging system. Returning to the case of 6.9 µm × 6.9 µm F–P cavity color filter, the bottom right part of the grayscale map for electron beam lithography is shown in Fig. 3a. From the microscope image which corresponds to the inset area (red dashed box) of Fig. 3a, we can find that the fabricated F–P cavities show good color filtering effects (Fig. 3b). The height profile of the randomly distributed F–P cavities is characterized by using the white light interferometer. As shown in Fig. 3c, the surface of each color filter is very smooth and the edges between the neighboring pixels are quite clear, suggesting the high quality of grayscale electron beam lithography.

**Figure 3.**
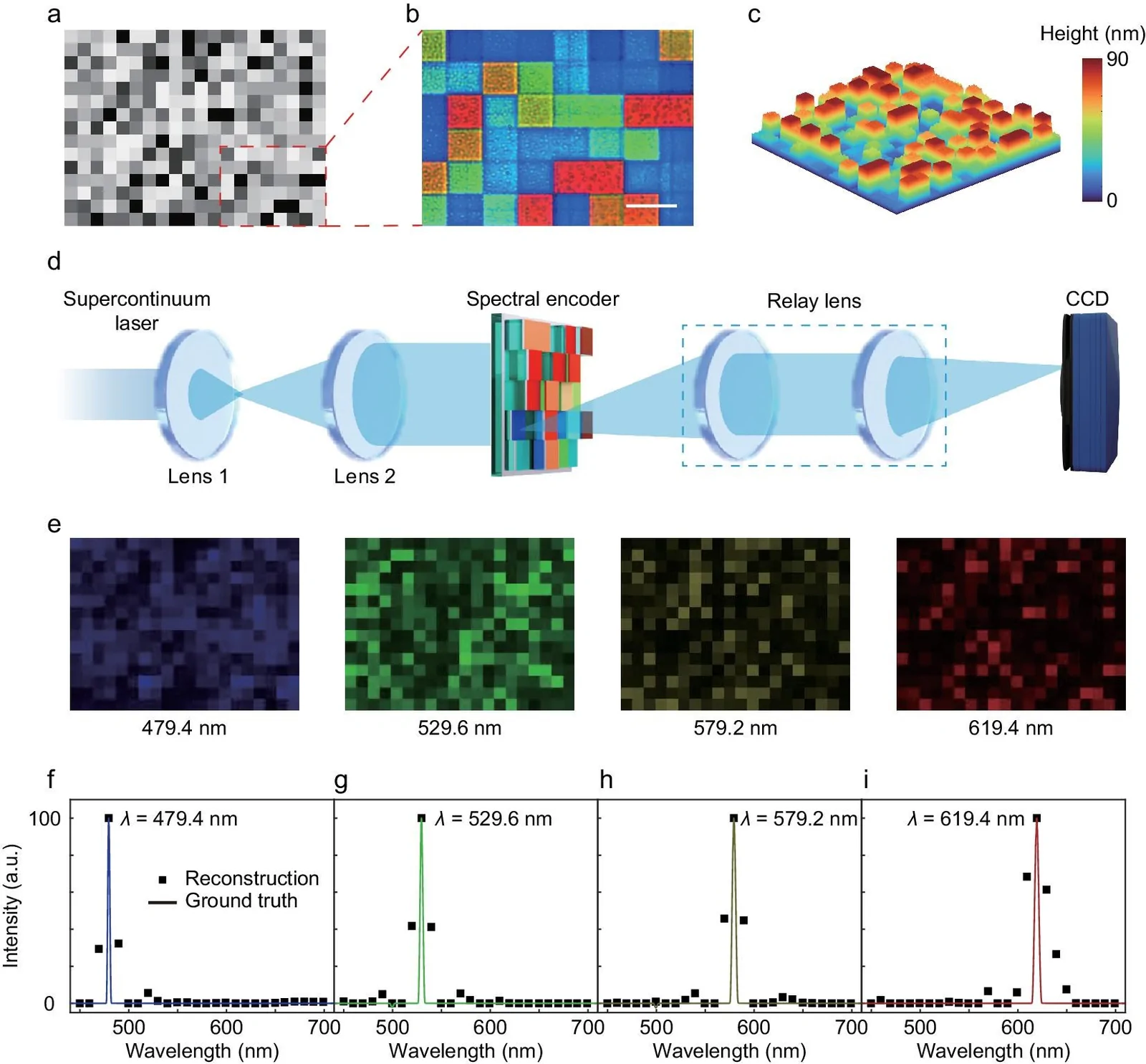
Calibration of the optical properties of the 6.9 µm × 6.9 µm F–P cavity color filter array. (a) The bottom right part of the grayscale map for electron beam lithography. (b) Microscope image of the randomly distributed F–P cavity color filters which are indicated by the red dashed box in (a). Scale bar: 10 µm. (c) Height profile of the F–P cavity color filter array measured with white light interferometer. (d) Optical setup for calibrating the optical properties of the F–P cavity color filter array (spectral encoder). The supercontinuum laser beam is expanded and used to illuminate the F–P cavity color filter array. The relay lens is used to project the image of the randomly distributed color filters to the monochrome CMOS sensor with a 1:1 magnification ratio. The integration time of the CMOS sensor is 2.0 s for all the laser wavelengths. (e) Four slices of the measurement matrix at wavelengths of 480, 530, 580 and 620 nm. (f–i) Reconstructed spectra of the incident laser at four typical wavelengths. The solid squares represent the reconstructed spectra (weight coefficient *τ* = 0.7), and the solid lines are the ground truth spectra of the incident light laser measured by using the commercial spectrometer (USB2000, Ocean Optics).

Firstly, a calibration system schematically illustrated in Fig. 3d is designed to experimentally characterize the measurement matrix of the F–P cavity color filter array. In this system, the randomly distributed color filters are 1:1 projected to a monochrome CMOS sensor (CS165MU, Thorlabs) by using a relay lens (Edmund Optics) with focal length of 45 mm and numerical aperture (N.A.) of 0.2676. The spectral response of the color filter array is characterized with a supercontinuum laser source (NKT Photonics). The intensity and wavelength of the output light are controlled by an acousto-optic tunable filter. After passing through an integrating sphere (ISP-30–6-R, Ocean Optics), the coherence of the laser beam is greatly reduced. Then, the light beam is collimated by a telescope assembly and normally incident onto the color filter array. By varying the laser wavelength from 450 to 700 nm at a step of 10 nm, the transmittance of all the randomly distributed color filters is recorded in 26 monochrome images. The intensity values in these images form as a measurement matrix for hyperspectral imaging after taking into account the laser power and integration time of the CMOS sensor. Figure 3e shows the fake-color images of the color filters under the light illuminations at four specific wavelengths (480, 530, 580 and 620 nm). With the measurement matrix, we also test if the spectra of the incident laser can be reconstructed. From the results in Fig. 3f–i, it is shown that the peak wavelength of the reconstructed spectra agrees well with the ground truth which is measured by using a commercial spectrometer (USB2000, Ocean Optics). It should be noted that the bandwidth of reconstructed spectrum is slightly broader than that of the ground truth (Note S6). In principle, this could be improved by increasing the number of spectral channels of the color filters and reconstruction channels.

Then, the hyperspectral imaging capability of the 6.9 µm × 6.9 µm F–P cavity color filter array is experimentally verified. A Macbeth color checker chart (X-Rite) is illuminated by a white light light-emitting diode (LED) source (Effilux) and imaged by our hyperspectral imaging system. A 16-mm-fixed focal length lens (Edmund Optics) with N.A. ∼0.04 is used to project the image of the color checker chart onto the F–P cavity color filter array and then relayed to the monochrome CMOS sensor (CS165MU, Thorlabs). As shown in Fig. 4a, the ground truth of the color checker chart is captured by using a color camera (CS165CU, Thorlabs) with the same sensor size as that of the monochrome one. Then, the hyperspectral imaging information is reconstructed by using the captured monochrome images and the measurement matrix obtained with the supercontinuum laser. The reconstructed data cube consists of 532 × 712 pixels and 26 spectral channels ranging from 450 to 700 nm with 10 nm interval. The reconstructed spatial and spectral information is synthesized into the color images (Fig. 4b). It is clear that the reconstructed color images of the color checker agree well with the ground truth. The corresponding spectra of six typical color patches are shown in Fig. 4c. The mean squared error (MSE) between reconstructed spectra and ground truth of all color patches is 0.0031–0.0523 (Fig. S9). The reconstructed images of the six color patches in different wavelength channels are shown in Fig. 4d (Note S7).

**Figure 4.**
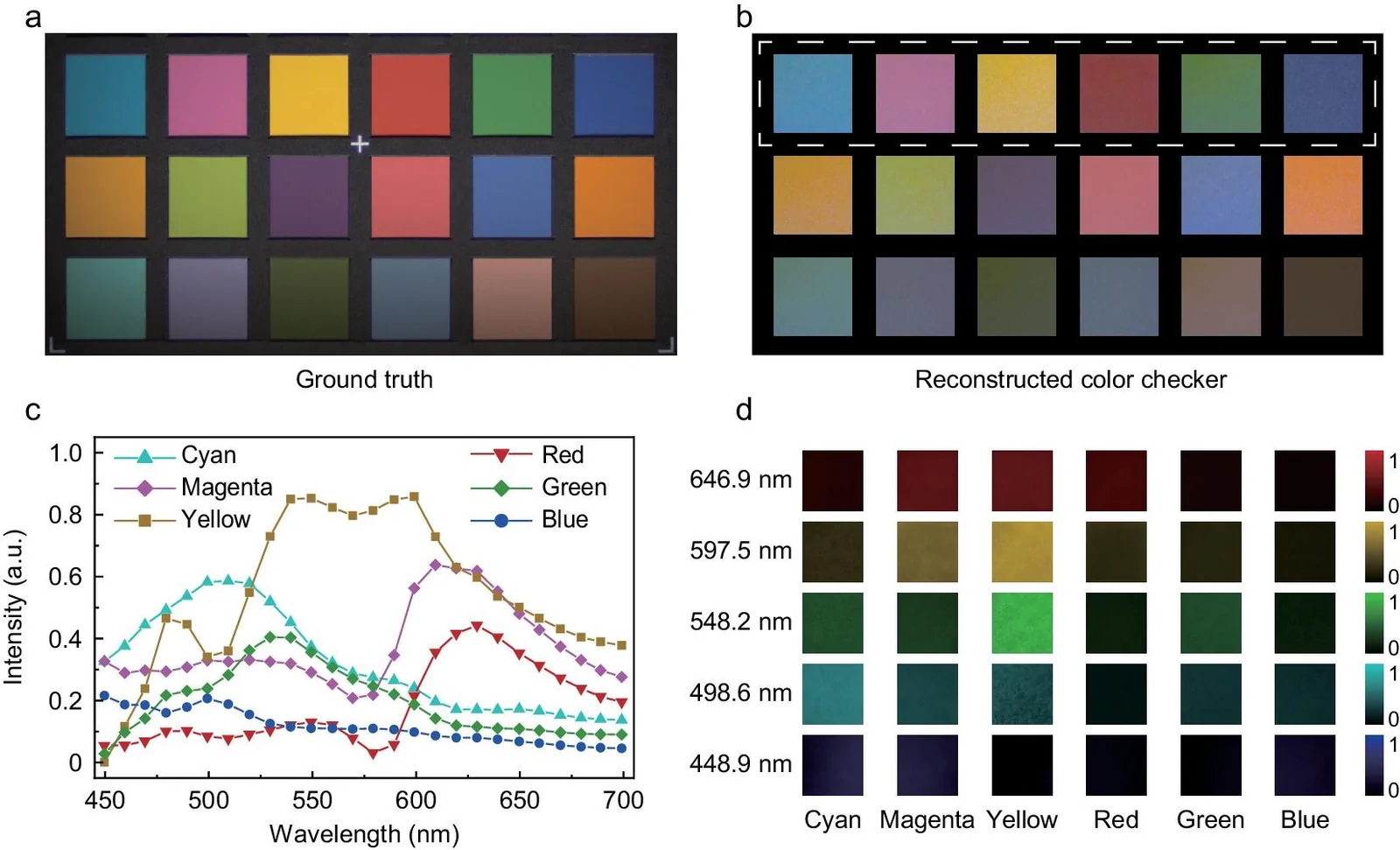
Hyperspectral imaging with the 6.9 µm × 6.9 µm F–P cavity color filters. (a) Ground truth of the Macbeth color checker classic mini. The ground truth is captured by using a commercial color camera (CS165CU, Thorlabs). (b) Reconstructed hyperspectral images (weight coefficient *τ* = 0.8). The RGB value of color patches is synthesized from the spectrum at each pixel on the monochrome CMOS sensor. (c) Spectra of the color patches indicated by the white dashed box in (b). (d) Intensity of the color patches at five distinct wavelength channels.

### Hyperspectral imaging with the megapixel F–P cavity color filters

To explore the spatial resolution limit of the proposed spectral encoder, we also design and fabricate the F–P cavity color filter array with pixel size of 5.2 µm × 5.2 µm and 3.45 µm × 3.45 µm, respectively. Figure 5a, b corresponds to the grayscale map and the microscope image of the randomly distributed color filters with pixel size 5.2 µm × 5.2 µm and pixel number of 1260 × 1000. From the transmission measurement results, it can be found that the peak wavelength of the 25 kinds of F–P cavities is perfectly located in the visible range (∼425–642 nm), showing a good color filtering effect (Note S8). Then, the hyperspectral imaging performance of the 5.2 µm × 5.2 µm color filter array is characterized with 32 spectral channels. As shown in Fig. 5c, d, six patches with red, yellow, blue, green, cyan, and magenta color are successfully reconstructed, exhibiting good consistency with the ground truth. Due to the difficulty in finely aligning the 5.2 µm × 5.2 µm F–P color filter array to the CMOS sensor, there is some deviation between the performance of hyperspectral imaging and the ground truth results, compared with that of the 6.9 µm × 6.9 µm device (Notes S9 and S10). Then, the spatial resolution of the F–P cavity color filter is further pushed to 3.45 µm × 3.45 µm (Fig. 5e, f), and no obvious proximity effect of the electron beam exposure between two neighboring pixels is observed. The optical properties of the 25 kinds of F–P cavities in the color filter array are characterized by using a self-developed confocal imaging setup, from which we find that the narrowband filtering effect of the F–P cavities can be maintained. Due to the alignment difficulty of the high-resolution color filter array, only the spectral measurement capability of this device is experimentally explored. The supercontinuum laser source with 34 spectral channels is used to get the measurement matrix of the 3.45 µm × 3.45 µm color filter array. The measured and reconstructed spectra of the laser source at six wavelength channels are shown in Fig. 5g–l. It can be found that the reconstructed peaks agree well with the ground truth (Note S11). However, the bandwidth of the reconstructed spectra is wider than that of the measured ones by using a commercial spectrometer (USB2000, Ocean Optics). If the laser wavelength is finely adjusted by a value of ∼2.0 nm through the acousto–optic tunable filter, a small spectral shift can be resolved by both the commercial spectrometer and the 3.45 µm × 3.45 µm F–P cavity color filter array based spectrometer.

**Figure 5.**
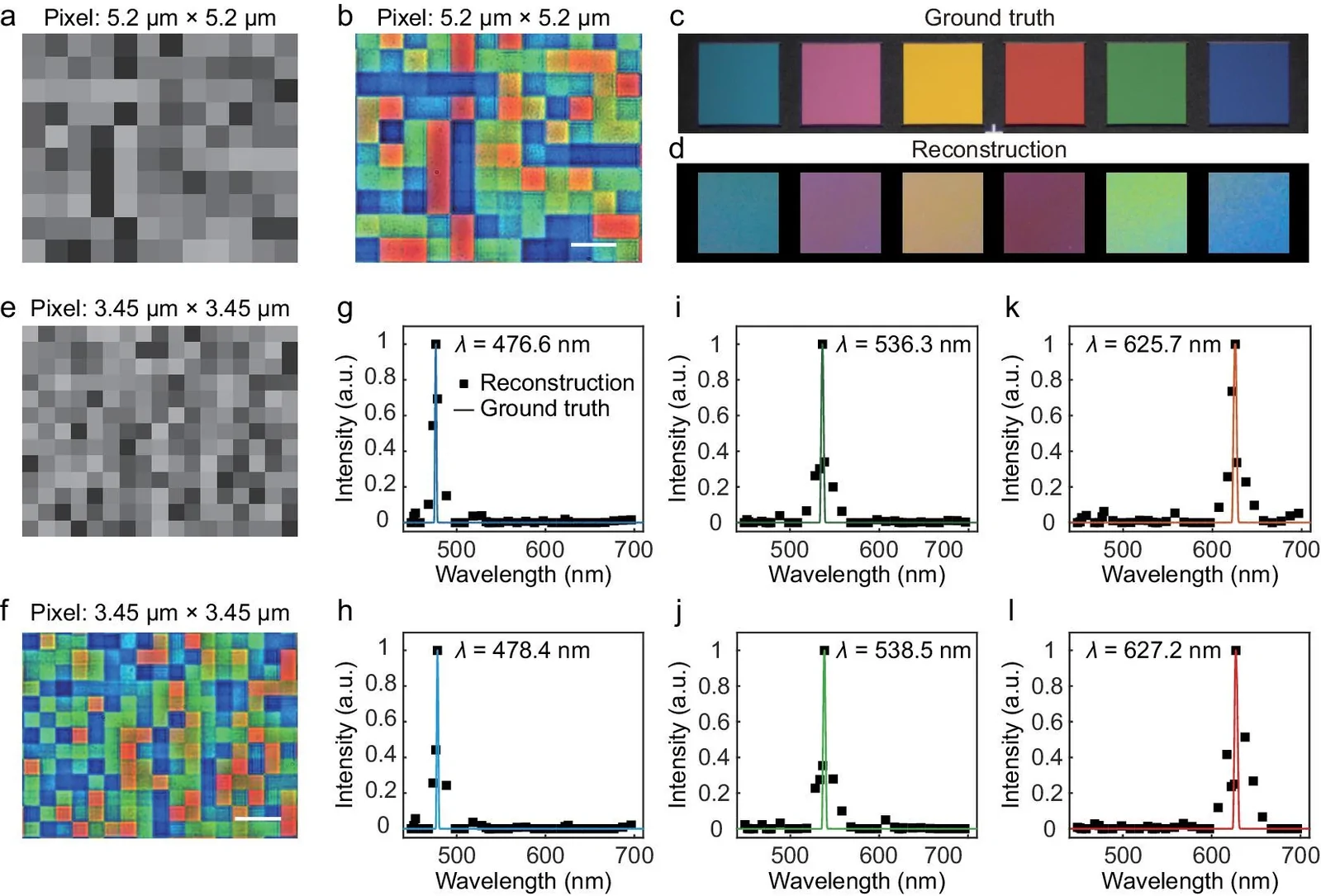
Hyperspectral imaging with the megapixel F–P cavity color filter array. (a) Bottom right part of the grayscale map of the 5.2 µm × 5.2 µm color filter array. (b) Microscope image of the randomly distributed F–P cavity color filters shown in (a). The pixel number of the color filter array is 1.26 million. (c and d) Ground truth (c) and reconstructed (d) images of the patches on the color checker (weight coefficient *τ* = 0.9). The hyperspectral imaging experiment is implemented with the 5.2 µm × 5.2 µm F–P cavity color filter array. (e) Bottom right part of the grayscale map of the 3.45 µm × 3.45 µm color filter array. (f) Microscope image of the randomly distributed F–P cavity color filters shown in (e). Scale bars in (b) and (f) are 10 µm. (g–l) Reconstructed spectra of the supercontinuum laser with the 3.45 µm × 3.45 µm color filter array. It is shown that two laser peaks with ∼2.0 nm spectral separation are successfully resolved. (g) and (h), (i) and (j), (k) and (l) correspond to the blue, green and red spectral regimes, respectively. The solid squares represent the reconstructed spectra (weight coefficient *τ* = 0.5), and the solid lines are the ground truth spectra of the incident light laser measured by using the commercial spectrometer (USB2000, Ocean Optics).

To gain deeper insight into the factors governing the reconstruction accuracy of the proposed hyperspectral imaging system, we examined the influence of the intrinsic bandwidth of the F–P cavity, as discussed in Note S12. Our analysis reveals that a wider F–P cavity bandwidth increases the inter-channel correlation of encoders, which in turn compromises the reconstruction accuracy. In addition, we have extended the discussion to include a detailed characterization of the spatial resolution and spectral resolution of the system, which are presented in Notes S13 and S14, respectively. On the spatial resolution front, the results indicate that decreasing the feature size of the F–P cavity leads to a marked improvement in spatial resolution. Consequently, our study of F–P cavities with pixel size of 3.45 µm × 3.45 µm offers a promising avenue for exploring the high-spatial-resolution hyperspectral imaging systems. Regarding the spectral resolution, we implement the spectral separation simulation under the dual-peak laser illumination, which demonstrates that the reconstructed spectral resolution is on the order of 60 nm. This finding underscores the necessity of adopting F–P cavity color filters with narrower bandwidth to achieve higher spectral resolution.

## CONCLUSION

In this work, we propose a strategy to realize hyperspectral imaging with the randomly distributed F–P cavity color filters, which consist of an Ag/PMMA/Ag sandwiched structure. The spacer thickness of the F–P cavities can be precisely controlled by one-step grayscale electron beam lithography process. We show that the F–P cavity color filter array with pixel size of 5.2 µm × 5.2 µm and pixel number of 1.26 million can be used to realize high-performance hyperspectral imaging. In addition, the spectral measurement with higher spatial resolution (pixel size: 3.45 µm × 3.45 µm) color filter array is experimentally demonstrated. Moreover, the structure we propose primarily relies on mature fabrication processes such as grayscale electron beam lithography and thin film evaporation, and no plasma etching process is involved. This makes our method inherently compatible with CMOS technology. For example, most of the commercial color CMOS sensors are manufactured by using photolithography and dye-doped photoresist [38–40]. The compatibility with the established manufacturing infrastructure makes our approach more promising for mass production. In the future work, the fabrication of the F–P cavity color filter array can be realized by using multi-beam electron beam lithography or ultraviolet laser direct writing, which may significantly reduce the time and cost of the fabrication while maintaining high spatial resolution. In principle, both the spectral and spatial resolution of the proposed hyperspectral imaging system can be greatly improved by directly integrating the F–P cavity array on the monochrome sensor. The grayscale lithography enabled metal–dielectric–metal F–P color filter array represents an advanced platform for high-performance hyperspectral imaging, and can be easily extended to different spectral regimes by appropriately choosing the corresponding materials and spacer thickness. Recent progress of optical metasurfaces with different structural configurations has significantly enhanced their capability for complex optical field manipulation [41–45]. By integrating metasurfaces and F–P cavity color filters, applications with strong spectral functionality can be realized, such as OLED displays with over 10 000 pixels per inch [46].

## METHODS

### Fabrication of the F–P cavity color filter array with grayscale electron beam lithography

The F–P cavity color filter is composed of two silver layers as mirrors and an electron resist layer as the spacer. A thin silicon dioxide layer is deposited on the top of the F–P cavities to reduce the oxidation effect of silver. Firstly, a ∼30-nm-thick silver layer is deposited on the fused silica substrate by using electron beam evaporation. Then, a ∼207-nm-thick electron resist (950 PMMA, MicroChem) is spin-coated on the silver layer and baked at 50°C for 10 min. The electron beam lithography technique is used to write the grayscale pattern on the PMMA layer with spatially variant exposure dose. The maximum electron dose applied to the resist is ∼246 µC/cm^2^. After the developing process, the second silver layer with a thickness of ∼30 nm and a 20-nm-thick silicon dioxide layer are deposited onto the PMMA pattern in sequence. Finally, the F–P cavity color filter array is obtained.

### Hyperspectral imaging experiment and image reconstruction

To conduct the hyperspectral imaging experiment, an LED-based white light source (Effilux) is used to illuminate the object (X-rite color checker classic mini). A 16-mm fixed-focal-length lens (Edmund Optics) is utilized as the imaging lens to project the image of the object onto the F–P cavity color filter array. Then, the image is relayed to the monochrome CMOS sensor with a 1:1 magnification ratio. In the subsequent imaging process, the white light for illumination is reflected by the objects. Then, the reflected light is coded by the randomly distributed F–P cavity color filter array. Since the raw images captured by the CMOS sensor only show the relative intensity of detected light, it would be an under-determined problem to analyze the spectral information of the objects. By using appropriate sparsity of the detected object, the backward reconstruction process can be conducted by minimizing the gradients between the pixels in the lateral directions. The details and schematic diagrams of the experimental setup are provided in Note S15. The TwIST algorithm is utilized to solve this problem in a self-developed MATLAB program. The iterative process is conducted to find the correct spectral information of the objects at specific positions. The synthetic RGB images are generated by calculating the CIE1931xyY values and RGB values of each pixel from the reconstructed spectra of the objects. All reconstruction algorithms are performed on a workstation equipped with an Intel (R) Core (TM) i7-8700 CPU @ 3.20 GHz, 64 GB. The simulation code is written in MATLAB. The time required for each reconstruction (532 × 712 pixels with 26 wavelength channels), including 500 iterations, is 940 s. The reconstruction is based on the standard TwIST algorithm. By applying neural networks and related algorithms, the reconstruction speed could be greatly improved [22,28,31,47].

## Supplementary Material

nwag526_Supplemental_File
