# Supplementary material for: Megapixel snapshot hyperspectral imaging with high resolution Fabry–Pérot color filter array
Source: Natl Sci Rev. 2026 Aug 22;13(20):nwag526. doi: 10.1093/nsr/nwag526 (PMC13702027; doi:10.1093/nsr/nwag526)
Supplement: Supplementary file 1 [file nwag526_supplemental_file.pdf]

Supplementary Data for

Megapixel snapshot hyperspectral imaging with high resolution Fabry–Pérot color filter array

Yu Chen[1,2,†], Jiaming Huang[1,†], Zixian Hu[1,†], Rong Rong[3], Qichang Ma[1], Peiqi Zeng[1], Jiafei Chen[3], Hoilam Tam[1], Junhong Deng[1], Kingfai Li[1], Kokwai Cheah[2] and Guixin Li[1,*]

[1]Department of Materials Science and Engineering, Southern University of Science and Technology, Shenzhen 518055, China.

[2]Department of Physics, Hong Kong Baptist University, Hong Kong 999077, China

[3]School of Integrated Circuits, Harbin Institute of Technology, Shenzhen 518055, China.

*Corresponding author. E-mail: ligx@sustech.edu.cn

†Equally contributed to this work

Contents

S1. Principle of hyperspectral imaging
S2. Numerical calculations and refractive indices of materials
S3. Optical characterization of PMMA thickness
S4. Optical setup for transmission spectra measurement
S5. Simulation of hyperspectral imaging process
S6. Reconstructed spectra of calibration laser (6.9-µm-pixel F–P cavity)
S7. Reconstructed images at different wavelength channels (6.9-µm-pixel F–P cavity)
S8. Single-pixel optical characterization
S9. Reconstructed spectra of calibration laser (5.2-µm-pixel F–P cavity)
S10. Reconstructed images at different wavelength channels (5.2-µm-pixel F–P cavity)
S11. Reconstructed spectra of calibration laser (3.45-µm-pixel F–P cavity)
S12. Spectral reconstruction error dependence on F–P cavity bandwidth
S13. Spatial resolution dependence on the pixel size of the F–P cavity
S14. Spectral resolution of F–P cavity-based hyperspectral imaging system
S15. Alignment process between the F–P color filter array and the CMOS sensor pixels
References

S1. Principle of hyperspectral imaging

In the imaging process, the incident light I with unknown spectra passes through the F–P cavity array and is recorded by a monochrome complementary metal–oxide–semiconductor (CMOS) sensor. For the ith pixel in the sensor, covered by the ith F–P cavity, the ith output value of the sensor image can be written as Equation (S1) [1,2]:

$$S_i = \int \frac{I(\lambda)T_i(\lambda)}{hc/\lambda} QE(\lambda) d\lambda, \qquad i = 1, 2, ..., m, \tag{S1}$$

where S_i is the detected signal in the sensor, I is the spectrum of an object, T_i is the transmittance of the corresponding F–P cavity, h is the Planck constant, c is the speed of light in vacuum, $QE(\lambda)$ is the quantum efficiency of the CMOS sensor, which means the ratio between converted electrons and the detected photons.

For simplification of the spectrum recovery algorithm, the interested wavelength is discretized into N bands. Then the unknown spectrum I is discretized into an N-dimensional vector, and Equation (S1) can be rewritten as:

$$S_i = \sum_{j=1}^{N} I_i(\lambda_j) M_i(\lambda_j), \text{ where } M_i(\lambda_j) = T_i(\lambda_j) QE(\lambda_j) \frac{\lambda_j}{hc}, \tag{S2}$$

where M is the measurement matrix, which is influenced by the transmittance of F–P cavities and the quantum efficiency of the CMOS sensor. In the simulated reconstruction, we calculate the measurement matrix M by multiplying the simulated transmittances of the F–P cavities and the quantum efficiency of the CMOS sensor from Thorlabs. In the experimental reconstruction, we obtain the measurement matrix by using the CMOS sensor to record the intensity distribution under the illumination of the monochromatic laser filtered by the F–P color filter array.

The reconstruction process is an ill-posed problem when only the captured monochrome image and the measurement matrix are known. We set a prior condition that the gradients of the reconstructed scene should be small, since natural scenes usually exhibit high sparsity. The reconstruction process can be presented by the cost function as Equation (S3):

$$\arg\min_I \left\{ \left\| S - \sum_{j=1}^{N} I(\lambda_j) M(\lambda_j) \right\|_2^2 + \tau \left\| \mathrm{TV}(I) \right\|_1 \right\}, \tag{S3}$$

where total variation TV is used to restrict the sparsity of the solution, τ is a coefficient to adjust the weight of the two terms. This equation is solved by using the Two-Step Iterative Shrinkage/Thresholding (TwIST) algorithm [3]. Then the hyperspectral images are reconstructed, providing both the spatial and spectral information.

S2. Numerical calculations and refractive indices of materials

To analyze the properties of F–P cavities, we build a three-dimensional model by using the commercial electromagnetic simulation software (Lumerical FDTD solution). This model consists of five layers: a bottom substrate, a bottom silver mirror, a PMMA spacer, a top silver mirror, and a silicon dioxide (SiO_2) encapsulation layer. As shown in Fig. S1a, the complex refractive index of silver is calculated from the Lorentz-Drude model [4], in which the oscillator strength f_1 is modified to 0.01. The complex indices of PMMA (Fig. S1b) and SiO_2 (Fig. S1c) are measured by using the spectroscopic ellipsometer (Horiba TF-UVISEL).

To balance the computing resource and accuracy, the mesh is set to 50 nm in the *x*-*y* plane and 1.0 nm in *z*-direction. The perfectly matched layer (PML) boundary condition is adopted in the propagation direction.

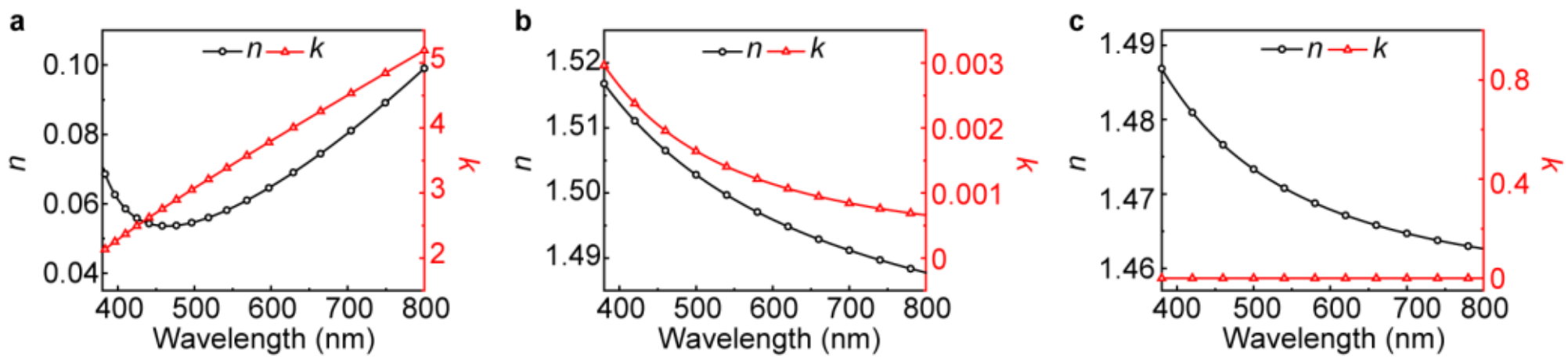

Figure S1. Refractive indices of materials. (a) Refractive index of silver. (b) Refractive index of PMMA. (c) Refractive index of silicon dioxide.

The thickness of the silver and PMMA layers has different effects on the optical performance of the F–P cavity. To identify the optimal configuration of the F–P cavities, we conduct a thickness sweeping for these two kinds of materials. Firstly, we change the silver thickness and calculate the corresponding transmission spectra while keeping the PMMA thickness at 92 nm, 119 nm, and 152 nm, respectively. The results, shown in Fig. S2, indicate that the silver thickness significantly influences both the bandwidth and the transmission efficiency. Increasing the silver thickness reduces efficiency. Conversely, decreasing the silver thickness broadens the bandwidth, which reduces the color purity of the transmitted light. To balance efficiency and bandwidth, we choose silver thickness of 25 nm. In addition, the SiO_2 layer has a minor impact on the device performance, serving primarily as an encapsulation layer. To simplify the design and fabrication process, the SiO_2 layer thickness is fixed at 20 nm.

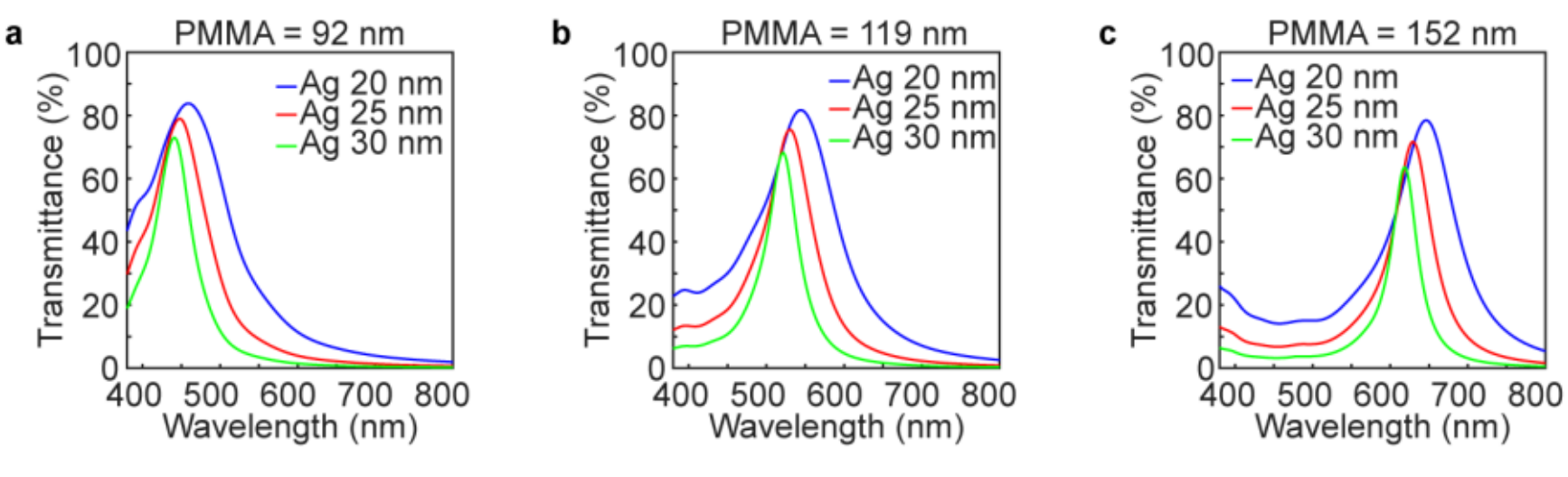

Figure S2. Calculated transmission spectra of the F–P cavities with varying silver layer thickness (20 nm–30 nm). (a–c) Thickness of PMMA layer is 92 nm, 119 nm, and 152 nm, respectively.

The thickness of the PMMA spacer varies from 80 nm to 180 nm to investigate the transmission spectra. Figure 2a in the main text shows the simulated transmission spectra of the F–P cavities with PMMA thickness ranging from 80 nm to 180 nm. Figure 2b shows 16 selected curves from Fig. 2a, corresponding to PMMA thickness of 84, 89, 95, 102, 105, 113, 120, 128, 137, 143, 149, 153, 160, 167, 172, and 177 nm, respectively. As the thickness of PMMA layer decreases, the central wavelength of transmitted light shifts from red to blue.

As the effective optical path length in the F–P cavities varies with incident angle, the transmission spectra have angular dispersion. Therefore, we perform FDTD calculations under different incident angles to quantify the angle-dependent optical properties of the cavities. A broadband plane wave is used as the light source, and the F–P cavity consists of the silver mirror layers (thickness: 25 nm) and the PMMA spacer (thickness: 92 nm, 119 nm, and 152 nm). For the case of oblique incidence, the broadband fixed angle source technique (BFAST) is employed. In the calculated results under oblique incidence with TM (Fig. S3a–c) and TE (Fig. S3d–f) polarization states, a slight blue shift (< 20 nm) of the peak wavelength can be observed as the incident angle increases from zero to 15 degrees, while the bandwidth remains nearly unchanged.

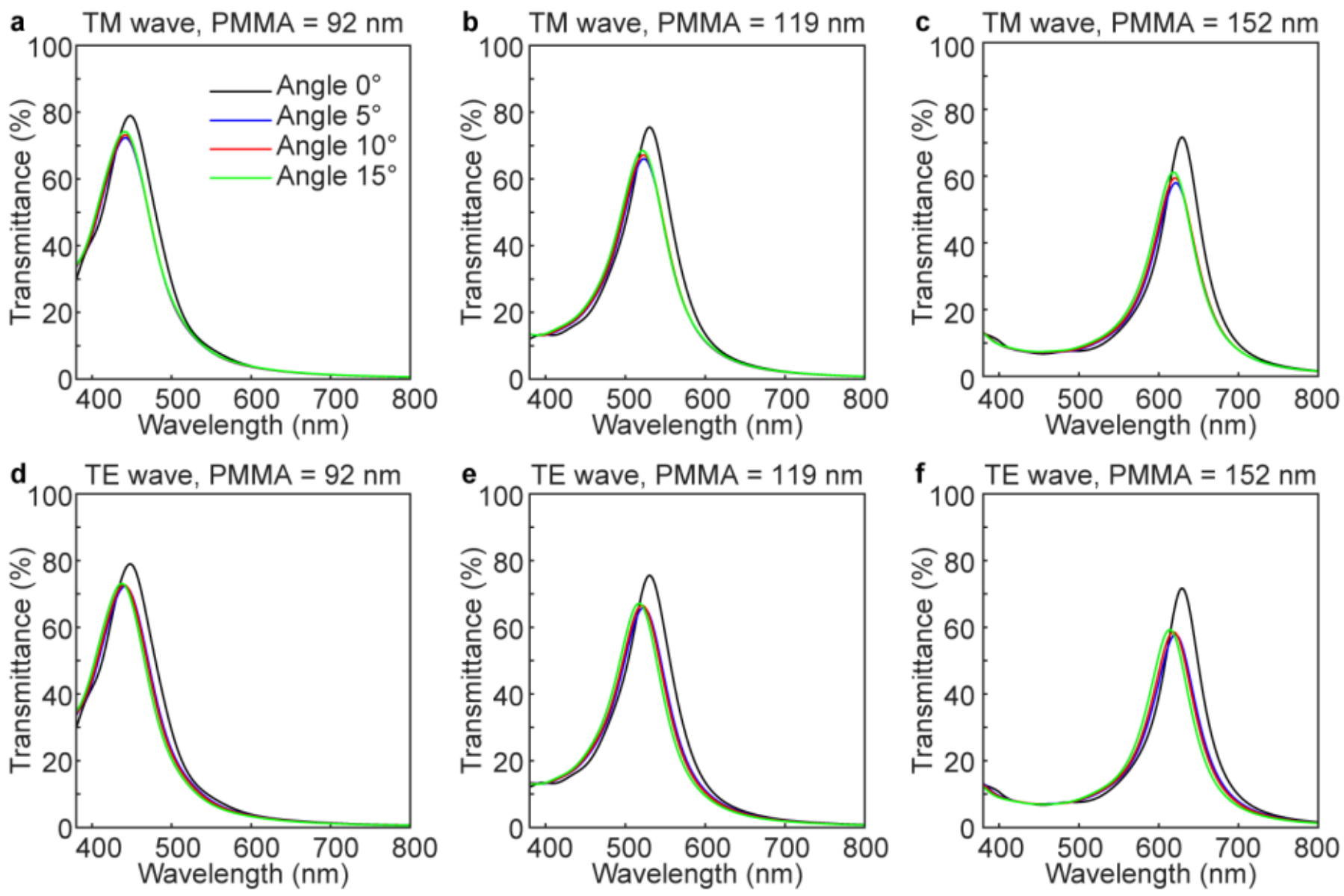

Figure S3. Calculated transmission spectra of F–P cavities under oblique incidence with TM (a–c) and TE (d–f) polarization states. The incident angle is set to be 0°, 5°, 10°, and 15°, respectively.

S3. Optical characterization of PMMA thickness

The height profile of color palettes is measured by using the white light interferometer (Zygo New View 9000). As shown in Fig. S4, the black dashed boxes indicate the reference plane in the sample, which is unexposed by the electron beam. The thickness of each palette in the main text is obtained from the difference between the measured thickness of the PMMA layer (after developing) and the measured depth of the F–P cavities.

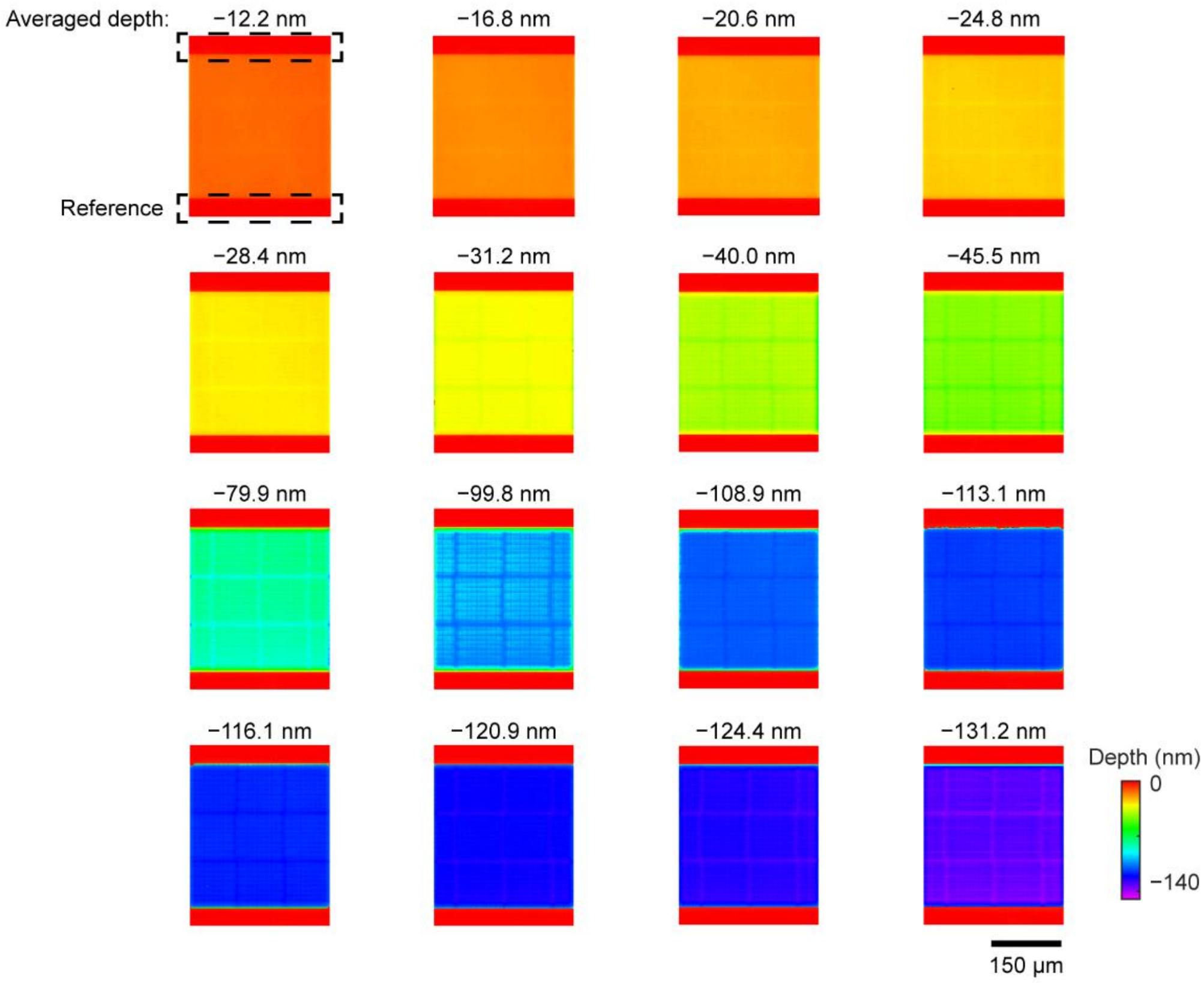

Figure S4. Height profile of the color palettes. The red regions (unexposed) surrounding each color palette serve as the reference surface (black dashed boxes) for white light interferometry measurement. As the exposure dose increases, the depth of the color palette increases.

S4. Optical setup for transmission spectra measurement

The transmission spectra of the F–P cavities are measured by using a home-made optical system shown in Fig. S5. The white light from a Xenon lamp (XLS400, Thorlabs) passes through a linear polarizer (horizontal direction, H), then is focused onto the F–P cavities by a 10× objective lens (Olympus Plan N). Then, the transmitted light is collimated by the second objective lens, then passes through the second linear polarizer (horizontal direction, H), and is finally collected by an optical fiber-coupled spectrometer (USB2000, Ocean Optics). The measured transmission spectra are shown in Fig. 2d. The relation between the grayscale value and the transmitted light is revealed by mapping the design files with the measured transmission spectra.

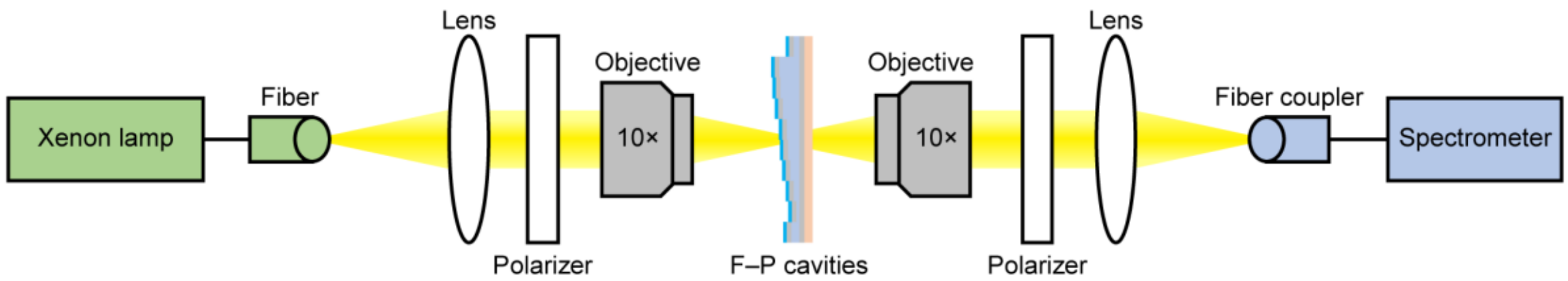

Figure S5. Optical setup for characterizing the optical properties of the F–P cavities. White light from a Xenon lamp is incident onto the F–P cavities, and the transmitted light is collected by an optical fiber-coupled spectrometer.

S5. Simulation of hyperspectral imaging process

According to the compressive sensing theory, the measurement matrix should be highly sparse. A set of F–P cavities is selected as the color filter array, which has 16 different spacer thickness, and the central wavelength of transmitted light covers 450 nm–700 nm. The simulated transmission spectra are used in the hyperspectral imaging process, as shown in Fig. S6a. The number is set to be the same for all kinds of cavities with different spacer thickness. All cavities are randomly distributed. The bottom right part of the simulated measurement matrix is visualized in four distinct wavelength channels according to the simulated transmission spectra of the F–P cavities (Fig. S6b).

To verify the spectral compressive effect of the randomly distributed F–P cavities, the imaging process is simulated with the hyperspectral images dataset ‘sponges_ms’ from the Columbia Imaging and Vision Laboratory (CAVE) [5]. We choose 26 images from the dataset, which contains the intensity information in the wavelength range from 450 nm to 700 nm with 10 nm as a step. The simulated signal from the CMOS sensor is shown in Fig. S6c. The reconstruction is conducted by using the TwIST algorithm as described in Note S1, where the weight coefficient τ is 2.0, and the iteration steps are 800, lasting for 1347 seconds.

There are two parameters that are used to evaluate the accuracy of the reconstruction results. Root mean square error (RMSE) is used to calculate the difference between the ground truth spectra and the reconstructed spectra of each pixel. It can be expressed as Equation (S4). Considering the RGB channels of all the pixels, the mean squared error (MSE) is also calculated with Equation (S5), which is then used to obtain the peak signal-to-noise ratio (PSNR) to show the quality of the reconstructed images, as expressed in Equation (S6).

$$\mathrm{RMSE}=\sqrt{\frac{\sum_{j=1}^{N}\left[I'(\lambda_j)-I(\lambda_j)\right]^2}{N}}, \tag{S4}$$

$$\mathrm{MSE}=\frac{\sum_{i=1}^{m\times n}\left\{\left[R_{origin}(i)-R_{recon}(i)\right]^2+\left[\mathrm{G}_{origin}(i)-G_{recon}(i)\right]^2+\left[\mathrm{B}_{origin}(i)-B_{recon}(i)\right]^2\right\}}{3\times m\times n}, \tag{S5}$$

$$\mathrm{PSNR}=10\times\log_{10}\left(\frac{255^2}{\mathrm{MSE}}\right), \tag{S6}$$

where N is the number of wavelength channels, and I' represents the reconstructed spectrum, while I represents the ground truth spectrum. R_{origin}, G_{origin}, B_{origin} represent the red, green, blue values of the ground truth image, while the R_{recon}, G_{recon}, B_{recon} represent the red, green, blue values of the reconstructed images. The pixel number of images is represented by m and n. The images are 8-bit data, so the maximum value is 255.

Compared with Fig. S6d, the synthesized RGB color of the reconstructed hyperspectral images (Fig. S6e) is close to the ground truth. The RMSE is calculated for each pixel and displayed as an error map (Fig. S6f). It can be observed that the edge of each color patch shows a larger error than that of the path center. The reason is that the gradient of the edge in the scene is large, and the sparsity is small. The maximum RMSE is 0.25, while the average RMSE of all

pixels is 0.0135. The PSNR of the reconstructed image is 86.2 dB. The reconstructed spectra of red, green, blue, and orange patches are detailed in Fig. S6g, which show high consistency with the ground truth. In conclusion, the simulated results indicate that the randomly distributed F–P cavities is an excellent spectral encoder.

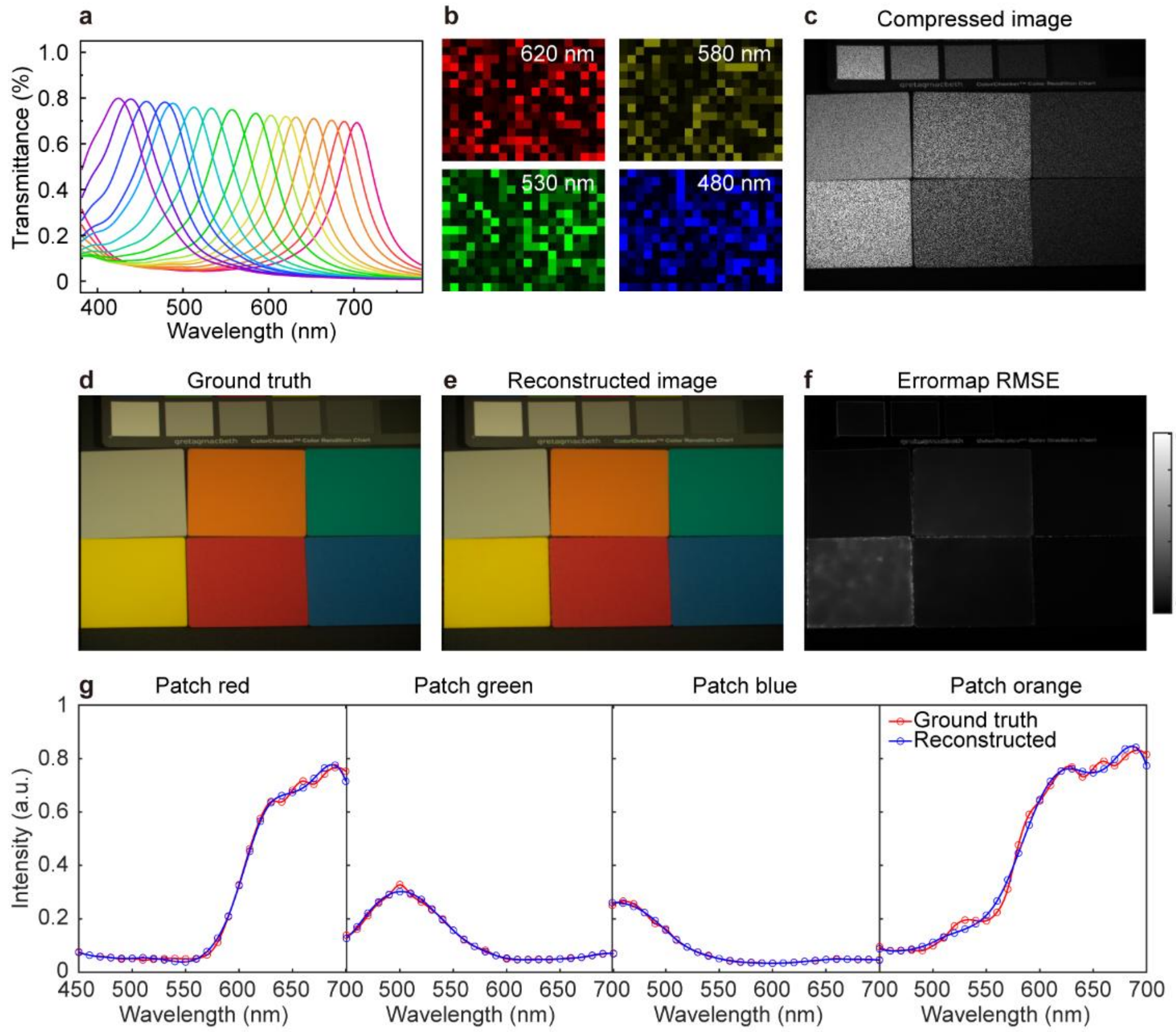

Figure S6. Simulation of the hyperspectral imaging process. (a) Calculated transmission spectra of the F–P cavities. (b) Simulated measurement matrix in four distinct wavelength channels. (c) Simulated monochrome image. (d) Ground truth of the hyperspectral dataset. (e) Reconstructed image in the hyperspectral imaging simulation. (f) Error map of the reconstruction results. (g) Comparison between the reconstruction and the ground truth spectra.

To validate the robustness of the hyperspectral imaging system, we select several hyperspectral images of real-world scenes from the CAVE database and reconstructed them individually. Fig. S7a displays the scene from the ‘flower_ms’ dataset, which features a composition of flowers. Fig. S7b shows the reconstructed RGB image, and Fig. S7c displays the reconstruction error map, where the maximum error is observed to be only 0.18. In Fig. S7d, the ground truth and reconstructed spectra of three representative points marked in Fig. S7a are compared, demonstrating excellent spectral fidelity across different spatial locations.

We further test the system on two additional datasets, ‘stuffed_toys_ms’ and ‘fake_and_real_peppers_ms’, with the corresponding results shown in Fig. S7e–h and Fig. S7i–l, respectively. In conclusion, these three sets of results demonstrate that our system achieves consistently high reconstruction quality across diverse materials and objects, confirming its robustness for practical hyperspectral imaging applications.

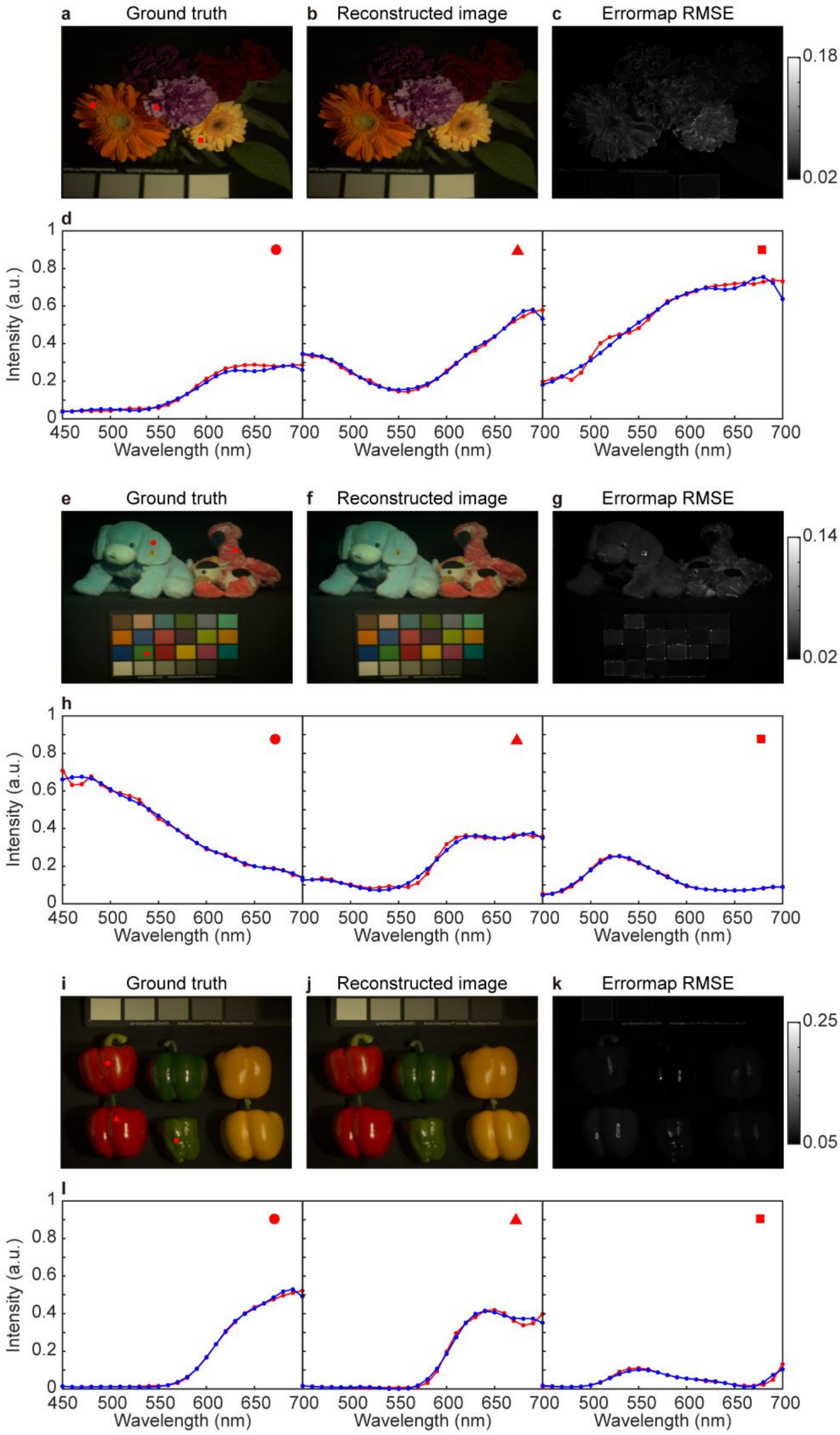

Figure S7. Robustness validation of the system using real-world scenes from the Columbia Imaging and Vision Laboratory (CAVE) database. (a) Ground-truth RGB image of the 'flower_ms' dataset. (b) Reconstructed RGB image. (c) Reconstruction error map, the maximum error is 0.18. (d) Ground truth and reconstructed spectra for the three marked points in (a). (e–h) Corresponding results for the 'stuffed_toys_ms' dataset. (i–l) Corresponding results for the 'fake_and_real_peppers_ms' dataset.

S6. Reconstructed spectra of calibration laser (6.9-µm-pixel F–P cavity)

The supercontinuum laser is incident into the hyperspectral imaging optical system to calibrate the measurement matrix of the system, as illustrated in Fig. 3d in the main text. The setting value of the wavelength channel of the supercontinuum laser varies from 450 nm to 700 nm with 10 nm as a step, while the central wavelength of the measured spectra is slightly different from the setting value. The wavelength channels of reconstruction are labeled with the central wavelength of the ground truth. Meanwhile, the captured monochrome images could be used to reconstruct the spectra of the incident laser. The reconstruction takes 20000 iterative steps with the weight coefficient τ = 0.7. Apart from the reconstruction results at four typical wavelength in Fig. 3e, more results are shown in Fig. S8, indicating that the fidelity of reconstructed spectra decreases as the central wavelength approaches 700 nm. It may be caused by the low spectral modulation of the F–P cavity encoder at the corresponding wavelength.

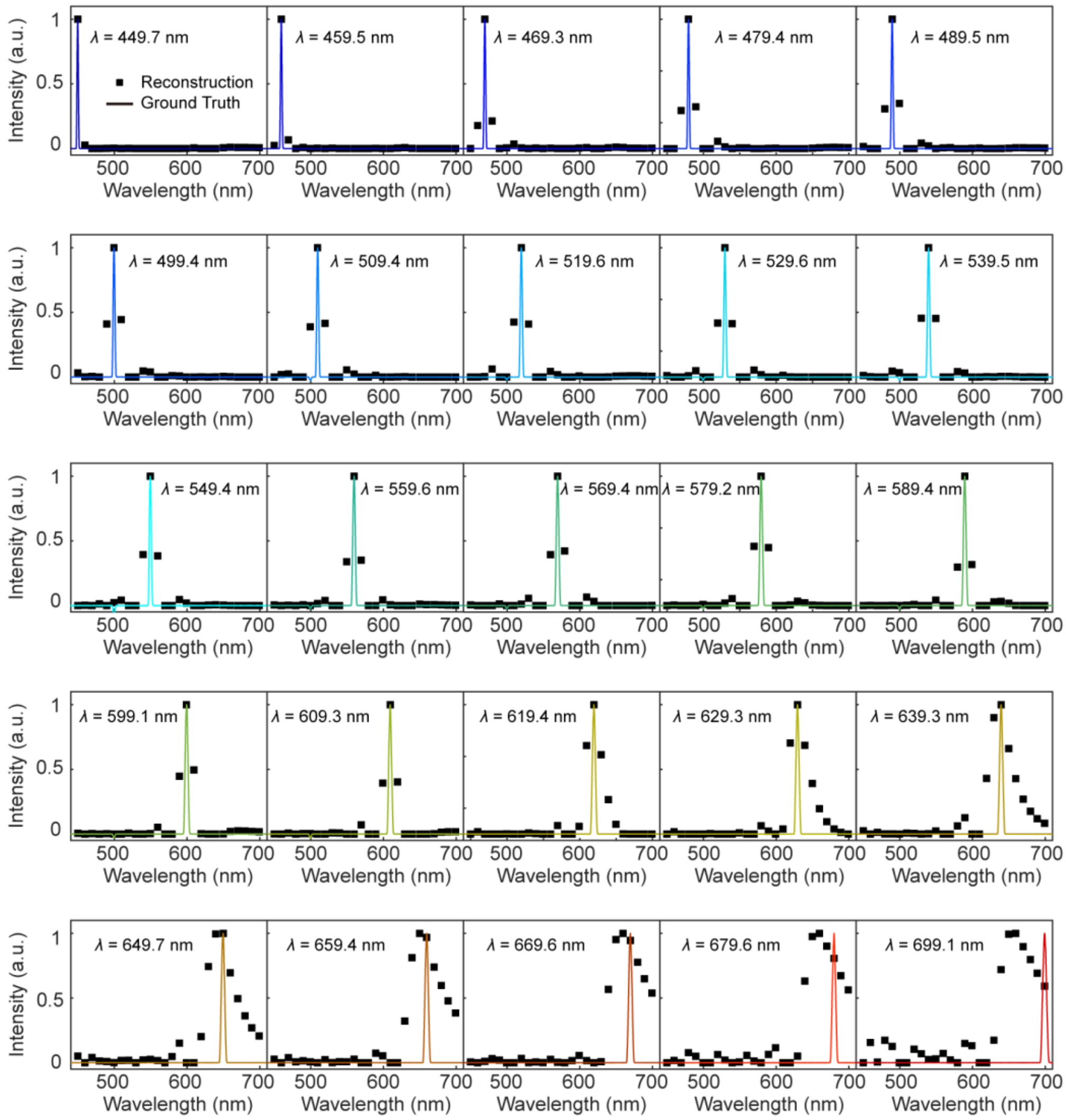

Figure S8. Reconstruction spectra of calibration laser with the 6.9-µm-pixel F–P cavity-based hyperspectral imaging system. Solid lines represent the ground truth measured by using a commercial spectrometer, while the solid squares represent the reconstructed spectra. The setting value of the wavelength channel of the supercontinuum laser varies from 450 nm to 700 nm with 10 nm as a step.

S7. Reconstructed images at different wavelength channels (6.9-μm-pixel F–P cavity)

The patches of the color checker are captured separately by using the 6.9-μm-pixel F–P cavity based hyperspectral imaging system. Then the spectra are reconstructed based on the methods described in Supplementary Note S1. The reconstruction takes 200 iterative steps with weight coefficient $\tau = 0.8$.

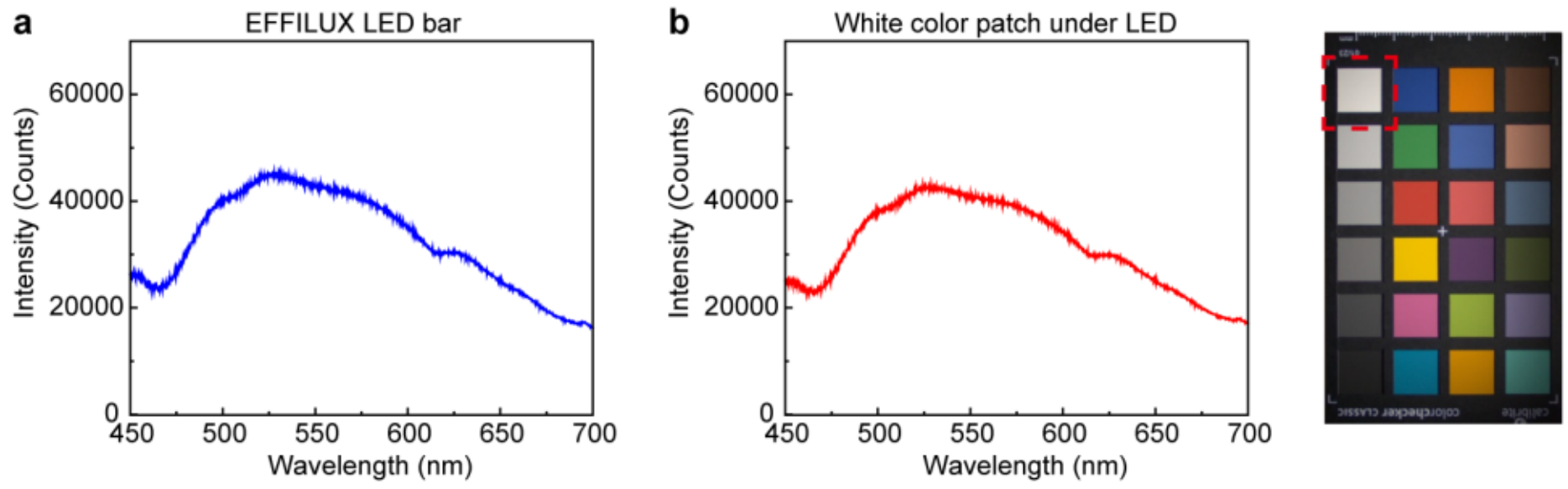

Figure S9. Measured spectra of the white light light-emitting diode (LED) source and the white color patch. (a) Measured spectrum of the white light LED source. (b) Measured spectrum of the white color patch under oblique illumination of the white light source.

Figure S9a shows the spectrum of the normally incident white light light-emitting diode (LED) source, which is measured by using a fiber-coupled spectrometer (USB2000, Ocean Optics) at a distance of about 75 mm from the light source. The white light is obliquely incident on the white color patch of the color checker, and the spectrum of the scattered light is measured as shown in Fig. S9b. It should be noted that the incident angle of ~ 60° is close to that of the illumination condition in the hyperspectral imaging experiments.

To eliminate the reconstruction error, spectrum corrections are applied. The spectral correction step can be described by the following Equations (S7) and (S8). Firstly, a spectral correction coefficient $C(\lambda)$ for each wavelength channel is calculated according to the measured and reconstructed spectra of the white color patch:

$$C(\lambda) = \frac{I_{\text{GroundTruth\_White}}(\lambda)}{I'_{\text{Reconstruction\_White}}(\lambda)}, \tag{S7}$$

where $I_{\text{GroundTruth\_White}}$ is the measured spectrum of the white color patch under the oblique illumination of the white light LED source (Fig. S9b); $I'_{\text{Reconstruction\_White}}$ is the reconstructed spectrum of the white color patch. Then, the reconstructed spectra are corrected based on the obtained spectral correction coefficient:

$$I'_{\text{Correct}}(\lambda) = I'_{\text{Reconstruction}}(\lambda) \times C(\lambda), \tag{S8}$$

where $I'_{\text{Reconstruction}}$ is the reconstructed spectrum, I'_{Correct} is the reconstructed spectrum after the spectral correction. To evaluate the reconstruction accuracy, the reconstructed spectra after spectral correction are compared with the ground truth spectra (Fig. S10). The ground truth spectra are obtained based on the spectrum of the white light LED source (Fig. S9a) while taking into account the measured reflectance of each color patch under normal incidence. Both the ground truth spectra and the reconstructed spectra after correction are normalized individually. As a result, the MSE of all the spectra is about 0.0031 ~ 0.0523, which is calculated as Equation (S9):

$$\mathrm{MSE} = \frac{\sum_{j=1}^{N}\left[I'(\lambda_j) - I(\lambda_j)\right]^2}{N}, \tag{S9}$$

where N is the number of wavelength channels, and I' and I represent the reconstructed and the ground truth spectra, respectively.

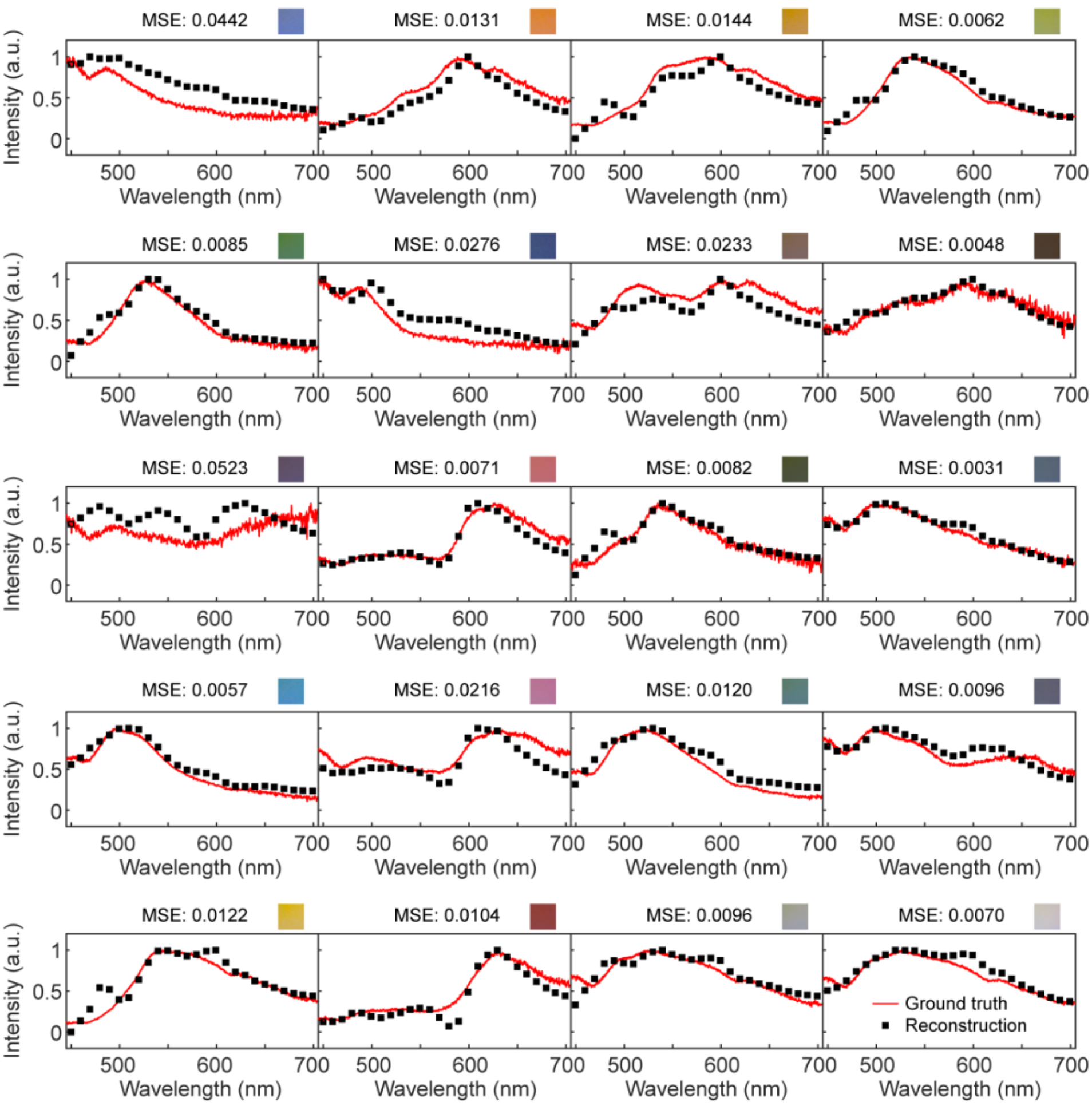

Figure S10. Comparison between the reconstruction and ground truth spectra. The reconstructed spectra after correction are normalized individually to compare with the ground truth spectra. The mean squared error (MSE) of the spectra of the 20 color patches is about 0.0031 ~ 0.0523.

To summarize, this correction is necessary since the regularization parameter applied to all the wavelength channels is the same in the TwIST iterative algorithm, while the measured transmittance of different wavelength channels varies. Therefore, without the correction, the convergence rate for each channel is affected by the transmittance difference, which is ultimately manifested as residual errors in the reconstructed spectra. To address this issue, the spectral correction procedure is implemented based on the reflectance spectrum of a standard white patch illuminated by a white light source (Effilux). We divide the theoretical white light spectrum by the reflected white spectrum to obtain a correction coefficient for each wavelength channel, and all the reconstructed spectra are subsequently calibrated using these coefficients. By calibrating against a standard white light reference, we obtain a set of correction coefficients

that are specific to this system. Importantly, the same set of coefficients can be applied to all the pixels of the hyperspectral image, regardless of the spatial location or the time of acquisition. As for the apparent large error observed in some curves in Fig. S10, it is due to the low intensity of the corresponding signals. The curves shown in Fig. S10 are normalized, which makes the visual appearance of the error more obvious, while the actual maximum MSE is only ~ 0.05.

The first column of Fig. S11 shows the captured raw images of different color patches. To compare the reconstructed images and the ground truth images, the reconstructed spectra after spectral correction (wavelength range: 450 nm–700 nm) are synthesized into RGB values through the CIE 1931 xyY color space. Since the wavelength range is smaller than the sensitive range of human eyes, RGB white balance is applied to all the synthesized images by calculating the RGB correction coefficient from the white color patch. Therefore, both the spectral and RGB correction are implemented in the reconstruction of color images. Synthesized RGB images are presented in the second column of Fig. S11, which are close to the ground truth. It can be observed that the intensity distributions of the images in different wavelength channels are distinctive (the third column to seventh column of Fig. S11). Moreover, there is no obvious distortion or noise in the reconstructed images. Therefore, the reconstruction has high fidelity in both spectral and spatial information domains. At present, the practical performance of our devices may be limited since the experimental setup is a proof-of-principle one, in which there is a relay lens between the F–P color filter array and the CMOS camera. Therefore, this configuration might not perform well in complex conditions containing both distant and nearby objects. In future research, we plan to integrate the F–P color filter array directly on the sensor of the CMOS camera and verify its hyperspectral imaging capability in more practical scenes.

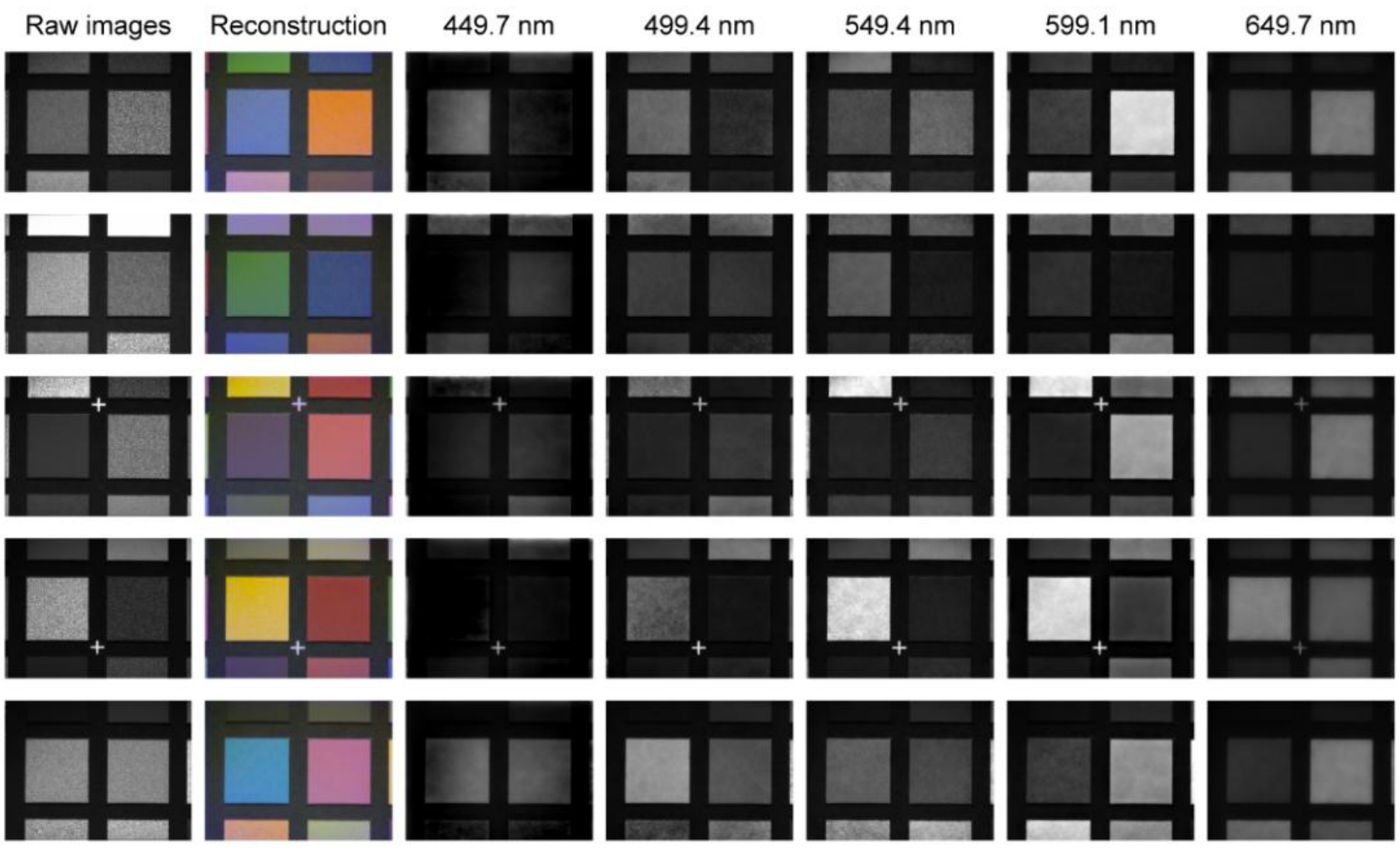

Figure S11. Reconstructed images with the 6.9-µm-pixel F–P cavity-based hyperspectral imaging system. Raw images of five different areas in the color checker are shown in the first column, which are obtained by using the 6.9-µm-pixel F–P cavity-based hyperspectral imaging system. The reconstruction results are synthesized into RGB images, as shown in the second column. The reconstructed images of five different wavelength channels show high fidelity in both spatial and spectral domains.

S8. Single-pixel optical characterization

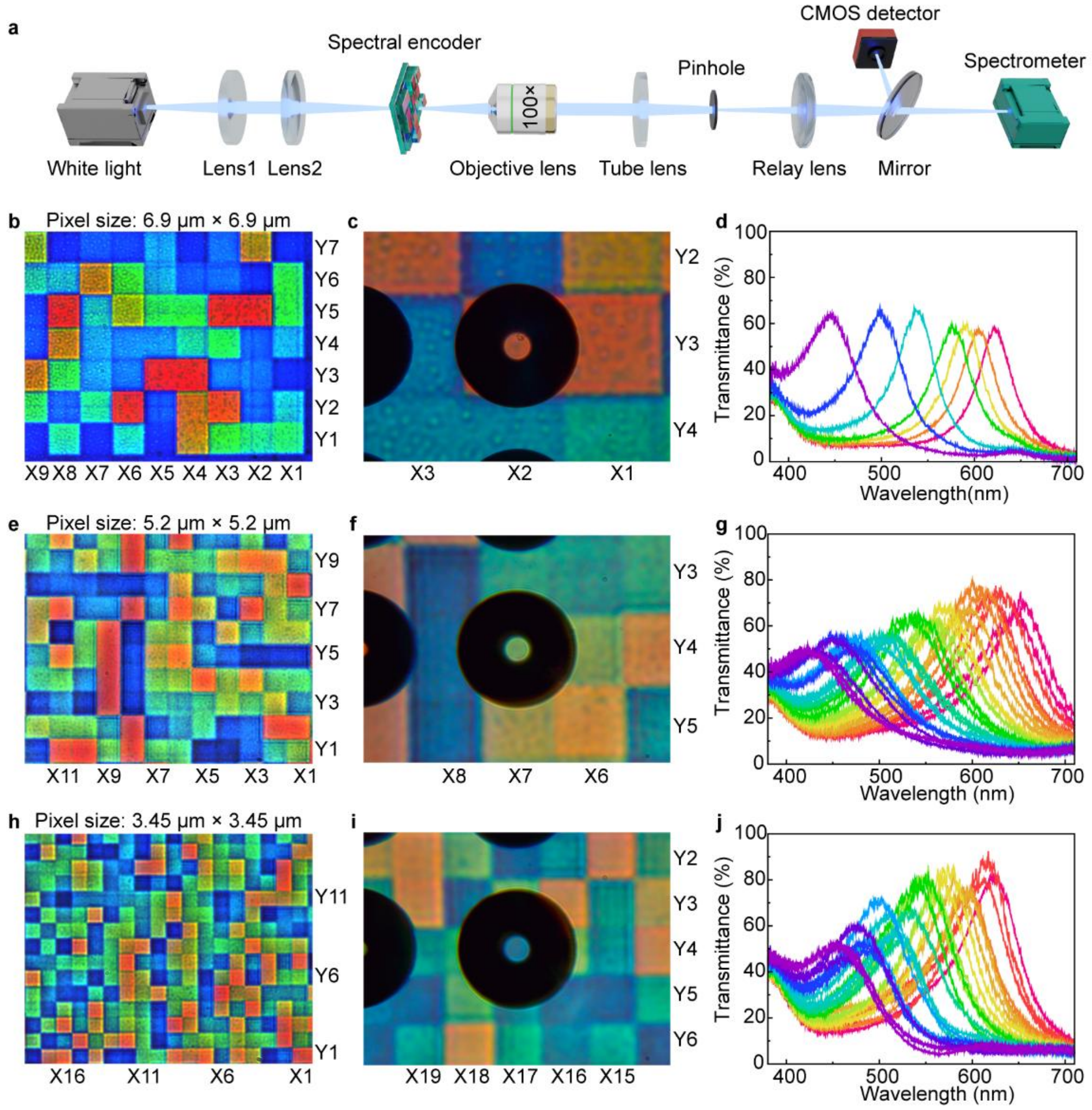

Figure S12. Single-pixel optical characterization. (a) Optical setup for single-pixel optical characterization. (b, e and h) Microscope images of the random color filter arrays with pixel size of 6.9 μm × 6.9 μm, 5.2 μm × 5.2 μm, and 3.45 μm × 3.45 μm, respectively. (c, f and i) The pinhole used to choose the area of measurement. (d, g and j) Measured single-pixel transmission spectra of the F–P cavities.

A micro-area transmission setup is utilized to characterize the optical property of the F–P cavity color filters at the single-pixel level (Fig. S12a). Light from a fiber-coupled Xenon lamp is collimated and focused on the F–P cavity, the transmitted light is collected by a high numerical aperture objective lens (N.A. = 0.8) and focused onto a pinhole by using a tube lens with focal length of 400 mm. The magnification ratio of the system is around 200. The fabrication process of the pinhole is as follows: A concentric circular ring with an outer diameter of 1.8 mm and an inner diameter of 400 μm is patterned on a photoresist layer by using direct laser writing photolithography. Then, a 180-nm-thick chromium (Cr) layer was deposited on the patterned resist by using electron beam evaporation. Finally, only the exposed area is covered by a layer of Cr after the lift-off process.

Color filter arrays with different pixel size (Fig. S12b,e,h) are characterized with the 'single pixel' optical system. The diameter of the pinhole is slightly smaller than that of the magnified image of the 3.45 μm × 3.45 μm pixel, as shown in Fig. S12c,f,i. Then, a 4f imaging system and a flip mirror are used to project the image of the pinhole to a color CMOS camera for position monitoring. When the flip mirror is put down, a fiber-coupled spectrometer (USB2000, Ocean Optics) is used to measure the transmission spectra of the color filters, as shown in Fig. S12d,g,j.

To evaluate the overall transmission efficiency of the F–P cavity array, we define the total transmittance T_{total} as the weighted sum of the spectrally integrated transmittance of each kind of cavity, normalized by the total incident energy. The total transmittance is described as:

$$T_{\text{total}} = \frac{\sum_{i=1}^{N} n_i \int_{\lambda_i^{\min}}^{\lambda_i^{\max}} T_i(\lambda) S(\lambda) d\lambda}{\sum_{i=1}^{N} n_i \int_{\lambda_i^{\min}}^{\lambda_i^{\max}} S(\lambda) d\lambda}, \quad \text{(S10)}$$

where the variables are defined as follows: $T_i(\lambda)$ is the experimentally measured transmission spectrum of the ith kind of F–P cavity; $S(\lambda)$ denotes the measured relative spectral distribution of the white light LED source (Effilux), as shown in Fig. S9a; n_i represents the number of the ith kind of F–P cavities, and $\lambda_i^{\min}$ and $\lambda_i^{\max}$ denote the corresponding spectral range; N is the total number of distinct F–P cavity types.

We estimate the total transmitted energy by integrating the experimentally measured transmission spectra. The transmission spectra of the F–P cavities used for this calculation are measured by using the optical setup in Fig. S3. As an example, the estimated total transmittance of the F–P cavity with pixel size of 5.2 μm × 5.2 μm is about 29.06%.

To explore the high-resolution color filter array, a periodic F–P cavity array with pixel size of 2.0 μm × 2.0 μm is fabricated and characterized. As shown in Fig. S13a, the microscope image demonstrates that the color filtering effect of the F–P cavities still exists, showing great potential in the field of high-resolution hyperspectral imaging. As shown in Fig. S13b, the height profile of the 2-μm-pixel color filter array is characterized by using the white light interferometer (Zygo New View 9000).

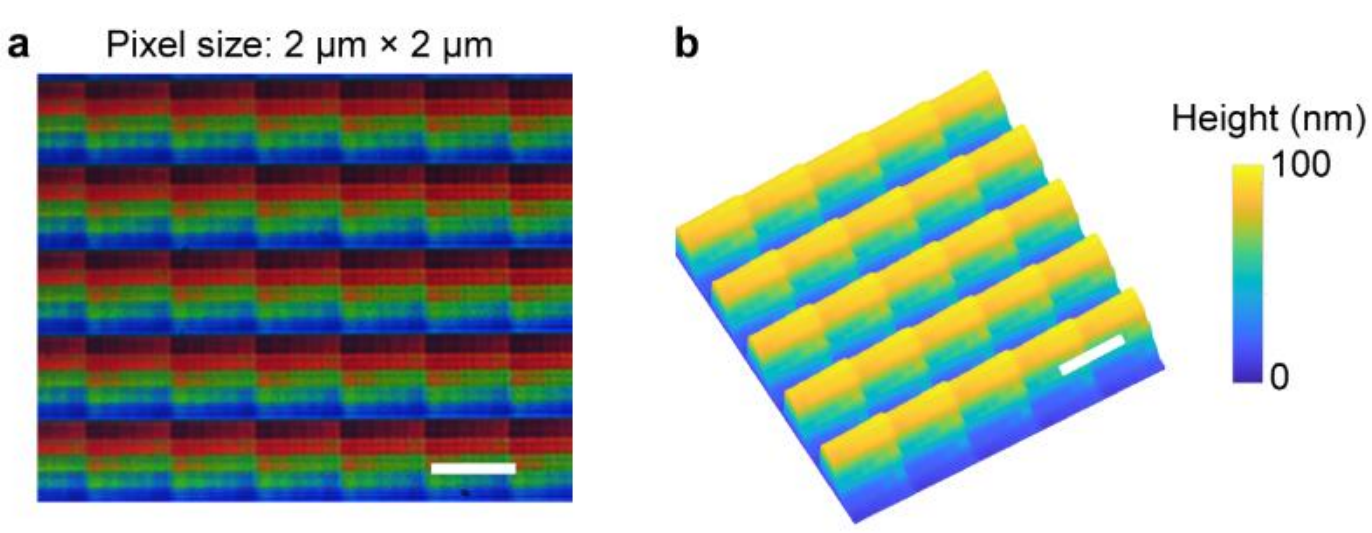

Figure S13. Characterization of the 2.0-μm-pixel F–P cavities. (a) Microscope image of periodic color filter array with pixel size of 2.0 μm × 2.0 μm. (b) Height profile of the color filter array with pixel size of 2.0 μm × 2.0 μm. Scale bar: 10 μm.

S9. Reconstructed spectra of calibration laser (5.2-µm-pixel F–P cavity)

The 5.2-µm-pixel F–P cavity-based hyperspectral imaging system is constructed and used to reconstruct the spectra of the supercontinuum laser. The reconstruction takes 30000 iterative steps with the weight coefficient $\tau = 0.2$.

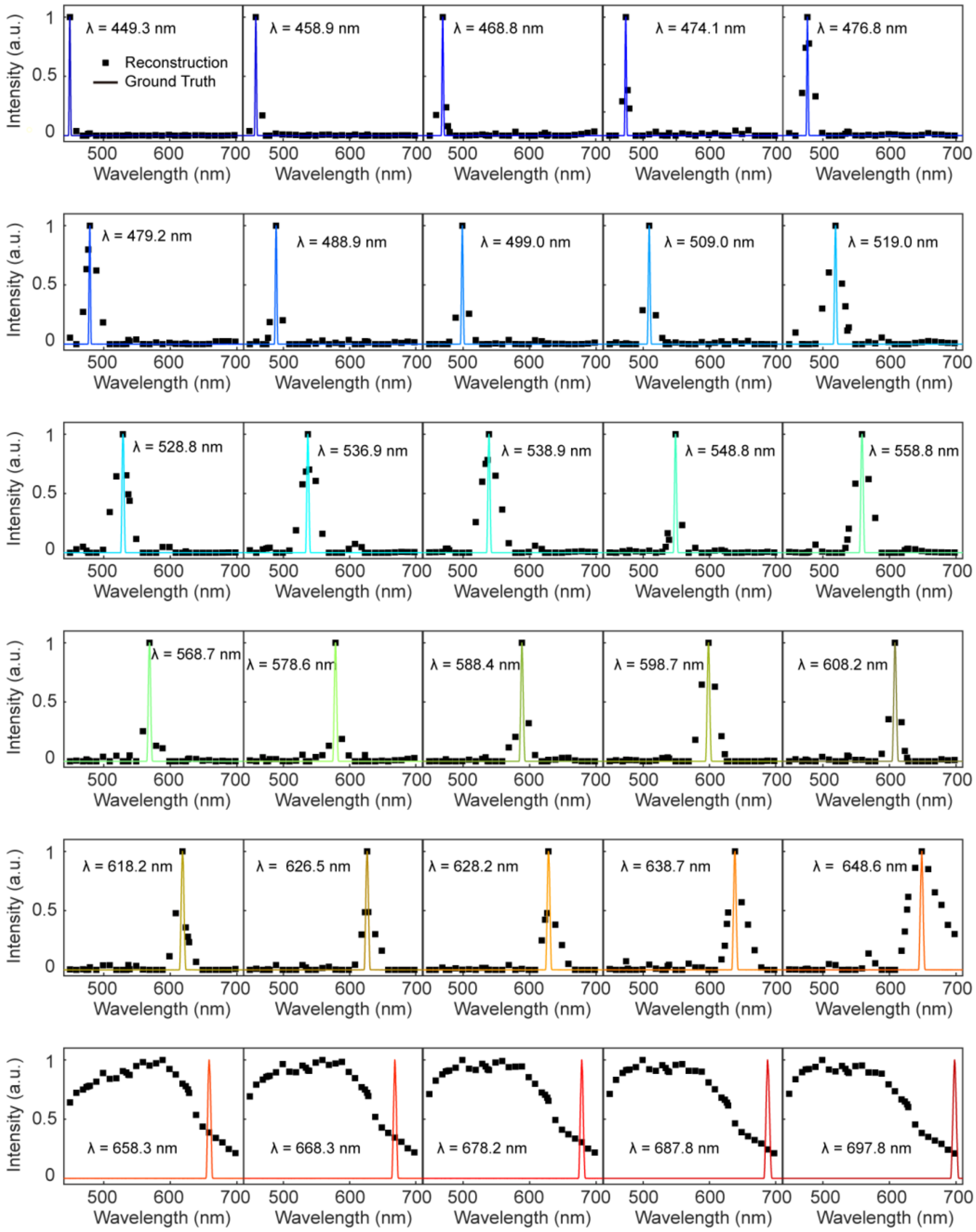

Figure S14. Reconstruction spectra of the calibration laser with the 5.2-µm-pixel F–P cavity based hyperspectral imaging system. Solid lines and solid squares represent the ground truth spectra and the reconstructed spectra, respectively. The setting value of the laser wavelength varies from 450 nm to 700 nm with 10 nm as a step. Six additional wavelength channels (setting value: 475 nm, 478 nm, 535 nm, 538 nm, 625 nm, and 628 nm) are added to verify the spectral peak sensitivity of the hyperspectral imaging system.

As shown in Fig. S14, the reconstructed results agree well with the ground truth at most wavelength channels. We also finely tune the shift of central wavelength about 2.0 nm through the acousto-optic tunable filter to verify the spectral peak sensitivity in the blue, green, and red regions. The reconstructed spectra deviate from the ground truth as the central wavelength approaches 700 nm, which may be caused by the low spectral modulation of the F–P cavity encoder at the corresponding wavelength.

S10. Reconstructed images at different wavelength channels (5.2-μm-pixel F–P cavity)

The hyperspectral imaging system is constructed based on the 5.2-μm-pixel F–P cavity encoder. The reconstruction takes 800 iterative steps with the weight coefficient τ = 0.9. The reconstructed images are synthesized into RGB images, which are very close to the ground truth of the color checker. The corresponding wavelength channels are shown in Fig. S15, demonstrating good reconstruction performance in both the spatial and spectral domains.

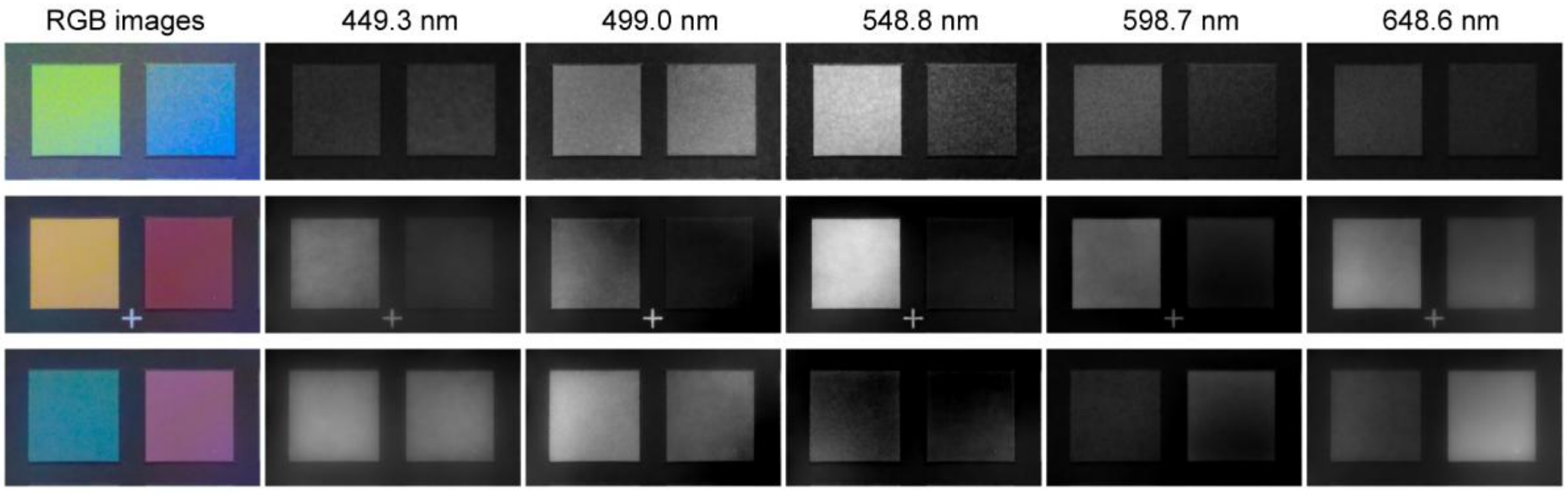

Figure S15. Reconstructed images with the 5.2-μm-pixel F–P cavity based hyperspectral imaging system. The reconstruction results are synthesized into RGB images in the first column. The reconstructed images of five different wavelength channels show high-quality reconstruction in both spatial and spectral domains.

The reconstructed spectra are compared with the ground truth, as shown in Fig. S16. The MSE is about 0.0057 ~ 0.1154. The reconstruction error is larger than that using the 6.9-μm-pixel F–P cavity based hyperspectral imaging system, which should be caused by the misalignment between the F–P cavities and the CMOS sensor pixels.

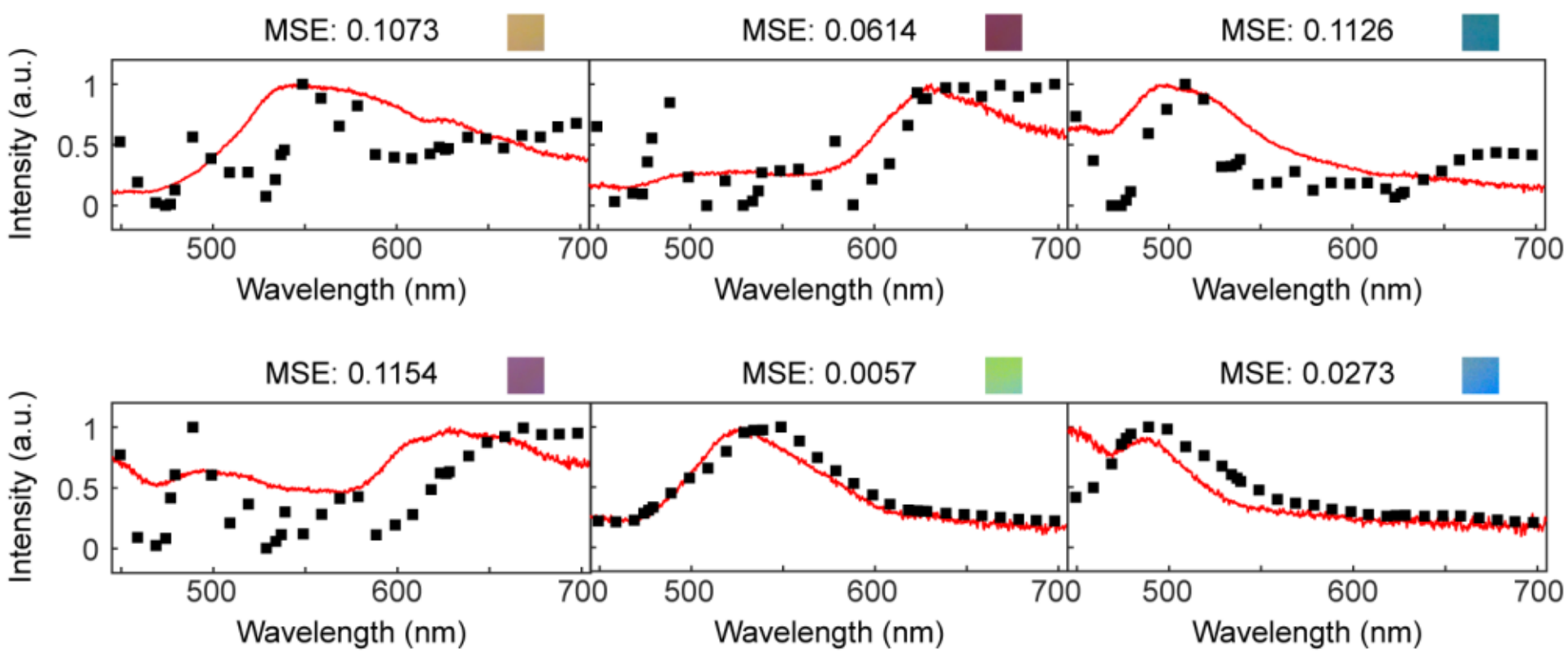

Figure S16. Comparison between the reconstruction and ground truth spectra of the color patches. The reconstructed spectra after correction are normalized individually to be compared with the ground truth spectra. The mean squared error (MSE) of the spectra of the 6 color patches is about 0.0057 ~ 0.1154.

S11. Reconstructed spectra of calibration laser (3.45-μm-pixel F–P cavity)

To validate the spectral peak sensitivity of the 3.45-μm-pixel F–P cavity based hyperspectral imaging system, the spectra of the light from the supercontinuum laser are reconstructed, where the central wavelength is tuned at the gap about 10 nm, 5.0 nm, 3.0 nm, and 2.0 nm. The reconstruction results at wavelength of 468.2 nm, 473.3 nm, 476.6 nm, and 478.4 nm demonstrate that a peak shift of 2.0 nm in the monochromatic laser input can be accurately reconstructed. The reconstructed spectra agree well with the measured ground truth in most cases except those of which the wavelength is longer than 667.0 nm, as shown in Fig. S17. The reconstruction takes 30000 iterative steps with the weight coefficient $\tau = 0.5$ for all wavelength channels except 458.7 nm and 568.1 nm, in which the weight coefficient $\tau = 0.2$ is set for better reconstruction.

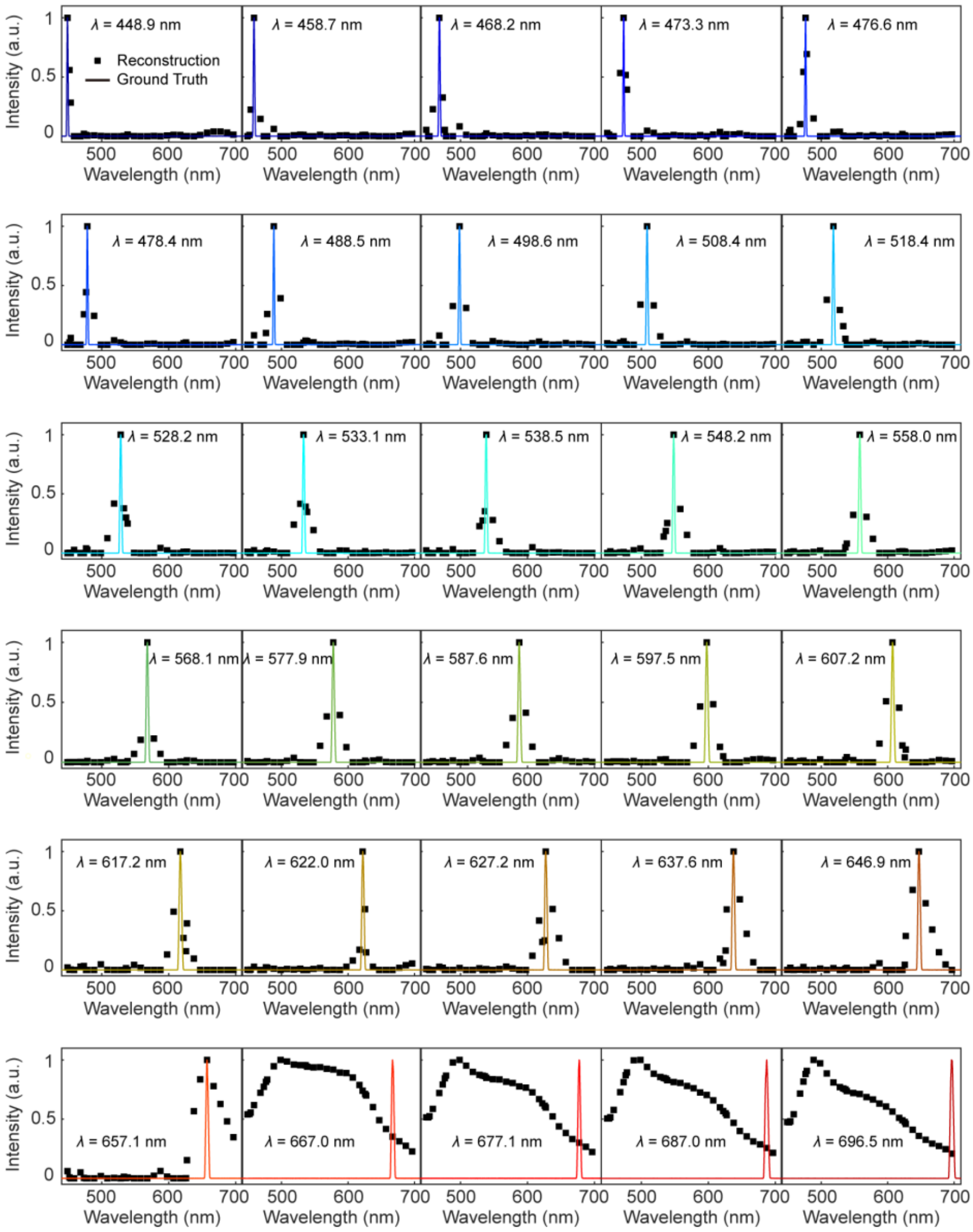

Figure S17. Reconstruction spectra of the calibration laser with the 3.45-μm-pixel F–P cavity based hyperspectral imaging system. Solid lines and solid squares represent the ground truth spectra and the reconstructed spectra, respectively. The setting value of the laser wavelength varies from 450 nm to 700 nm with 10 nm as a step. Eight additional wavelength channels (setting values: 452 nm, 455 nm, 475 nm, 478 nm, 535 nm, 538 nm, 625 nm, and 628 nm) are added to verify the spectral peak sensitivity.

S12. Spectral reconstruction error dependence on F–P cavity bandwidth

To investigate the spectral reconstruction error dependence on F–P cavity bandwidth, a set of analyses is conducted with FDTD simulations on different F–P cavity configurations.

F–P cavities with different silver layer thickness (20 nm, 25 nm, and 30 nm) are modeled as described in Supplementary Note S2. The transmission spectra that share the same peak wavelength but have different bandwidth are picked as the filter library, as shown in Fig. S18a–c. These transmission spectra are then mapped onto the F–P cavities layout according to their peak wavelength to construct distinct response matrices. Figure S18d–f presents the Pearson correlation matrices for the three kinds of color filter encoders.

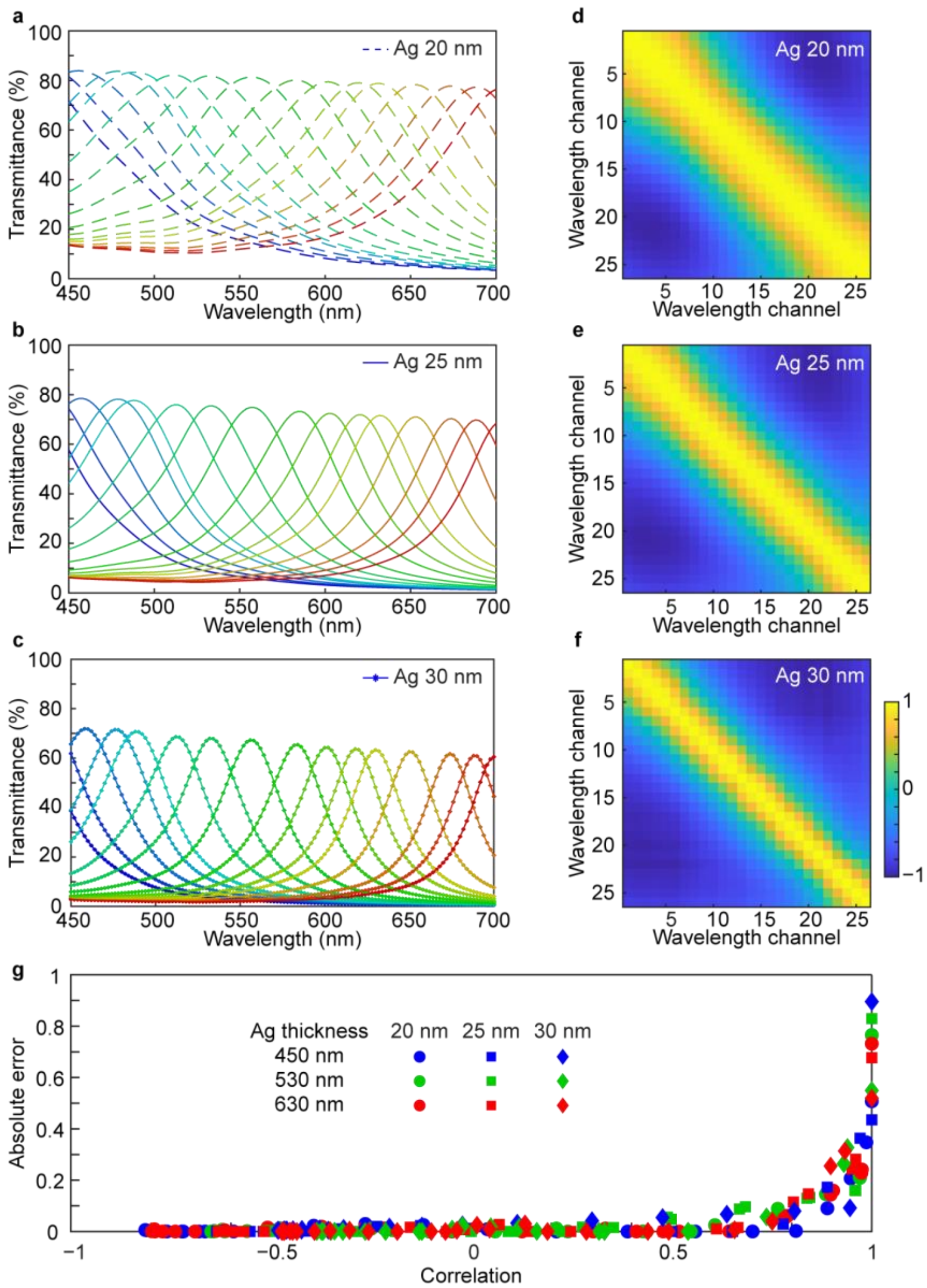

Figure S18. Relationship between the bandwidth of F–P color filters and the reconstruction error. (a–c) Transmission spectra of three representative F–P cavities with different bandwidth but identical peak wavelength (silver layer thickness: 20 nm, 25 nm, and 30 nm). (d–f) Inter-channel correlation maps of encoders composed of F–P cavities with different bandwidth. Channels 1–26 correspond to wavelength from 450 nm to 700 nm with a step of 10 nm. (g) Inter-channel correlation versus reconstruction error.

Assuming an input signal with an intensity of 1.0 in three specific wavelength channels (wavelength: 450 nm, 530 nm, and 630 nm) and an intensity of 0.0 in all other channels, we compress and subsequently reconstruct the signal by using the response matrices of the F–P color filters with different bandwidth, followed by the spectral reconstruction. Then, the absolute error between the reconstructed and the true incident spectra is compared. Figure S18g summarizes the results across three incident spectra, each paired with three kinds of encoders of different bandwidth, where we plot the pairwise channel correlations against the absolute reconstruction error. The results show that higher channel correlation leads to larger reconstruction errors. A comparison between the three panels reveals that the inter-channel correlation increases with broadening bandwidth, which in turn results in larger reconstruction errors. Therefore, we conclude that the bandwidth broadening of the F–P cavities elevate the inter-channel correlation, which in turn degrades the reconstruction accuracy, leading to a reduction in the effective spectral resolution of the reconstructed spectrum.

Meanwhile, we analyze the correlation of the experimentally acquired response matrix (6.9-μm-pixel F–P cavity array). It is found that the standard deviation (Fig. S19a) and variance (Fig. S19b) near the red channel (around 700 nm) are relatively small, indicating that the encoder's capability to modulate the incident light deteriorates in this spectral region, almost losing the signal compression functionality required for compressed sensing. Furthermore, the inter-channel correlation coefficients shown in Fig. S19c reveal that channels near 700 nm exhibit high correlation, which further degrades the accuracy of the reconstructed signal. These observations explain why the reconstruction error in the 700 nm band becomes larger, as shown in the spectral reconstruction results described in Supplementary Note S6.

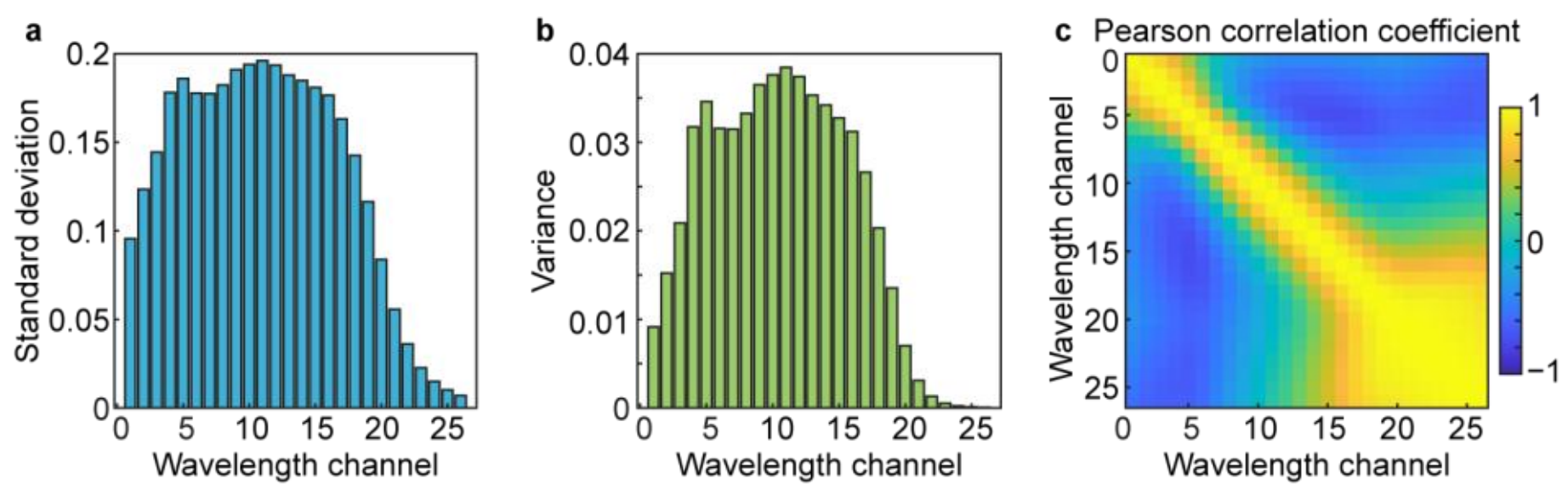

Figure S19. Channel analysis of the measured response matrix. (a) Standard deviation. (b) Variance. (c) Inter-channel correlation coefficients. Channels 1–26 correspond to the wavelength range from 450 nm to 700 nm with a step of 10 nm.

S13. Spatial resolution dependence on the pixel size of the F–P cavity

To investigate the pixel size influence of F–P cavity on the spatial resolution of the proposed hyperspectral imaging system, we conduct an analysis using a USAF 1951 resolution test chart. Seven groups from the resolution target are selected, and their linewidths are summarized in Table S1. These patterns serve as the test object, as illustrated in Fig. S20a. The image of the target consists of 512 × 512 pixels, with pixel size of 20 μm × 20 μm. In the simulation, the intensity values of the line patterns within the image are set to be 1.0 for each wavelength channel, while the background regions are assigned an intensity of zero across all wavelength channels. A randomly distributed F–P cavity array is generated, maintaining the same pixel dimension (512 × 512). The transmission spectra obtained from the FDTD simulations (Fig. S6a) are then matched with the random color filter array to form the response matrix, which is subsequently used for hyperspectral imaging simulations.

Table S1. Selected line groups from USAF 1951 resolution test chart.

Group	Element	Resolution (lp/mm)	Linewidth (μm)
1	5	3.17	157.7
1	6	3.56	140.4
2	1	4.00	125.0
2	2	4.49	111.4
2	3	5.04	99.2
2	4	5.66	88.3
2	5	6.35	78.7

In the simulations, the pixel size of the F–P cavity is set to be 20 μm × 20 μm, 10 μm × 10 μm, and 5 μm × 5 μm, respectively, while the arrangement and total number of F–P cavities remain unchanged. The change of the F–P cavity pixel size affects the field of view of the captured object, as indicated by the red dashed boxes in Fig. S20a. The reconstructed images are presented in Fig. S20b–d. The finest line group is magnified to compare the resolution of the reconstructed results, as shown in Fig. S20e–g. Comparing the three sets of reconstructed results, it is evident that when the characteristic size of the F–P cavity is 20 μm × 20 μm, the reconstructed line signals exhibit noticeable non-uniformity, indicating degraded reconstruction quality. This artifact diminishes as the cavity size decreases. It is revealed that an F–P cavity with smaller pixel size yields higher spatial resolution in the reconstructed hyperspectral images, which is consistent with the influence of pixel size on spatial resolution observed in conventional camera systems. This trend can be attributed to the dependence of compressed-sensing-based reconstruction on signal sparsity. The reduction of the F–P cavity pixel size will increase the spatial sampling rate, thereby enhancing signal sparsity and leading to superior reconstruction fidelity.

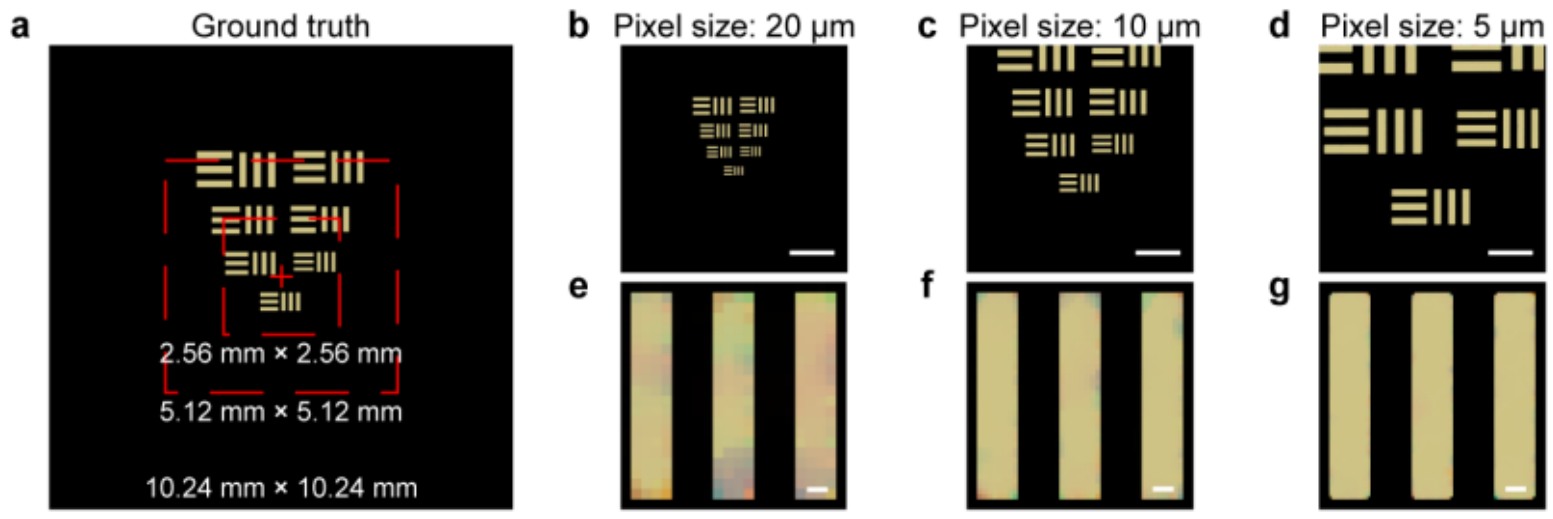

Figure S20. Influence of F–P cavity pixel size on the spatial resolution of the reconstructed hyperspectral image. (a) Ground truth of seven selected line groups from the USAF 1951 resolution test chart. The red dashed box indicates the field of view corresponding to different F–P cavity pixel size. (b–d) Reconstructed results under different F–P pixel size: 20 μm × 20 μm, 10 μm × 10 μm, and 5 μm × 5 μm, respectively. (e–g) Enlarged views of the finest line group (Group 2 Element 5) from (b) to (d), respectively, for comparison of spatial resolution. Scale bars in (b) to (d) are 2.0 mm, 1.0 mm, and 0.5 mm, respectively. Scale bars in (e) to (g) are 40 μm.

S14. Spectral resolution of F–P cavity-based hyperspectral imaging system

To rigorously assess the spectral resolution of the hyperspectral imaging system, we design a dual-peak separation simulation, which aims at evaluating the system's capability to distinguish closely spaced spectral features. Firstly, the output spectra of the incident supercontinuum laser source are characterized using a fiber-coupled spectrometer (USB2000, Ocean Optics), as shown in Fig. S21a, confirming a well-defined quasi-monochromatic line shape. Two Gaussian-shaped laser spectra with peak wavelength of λ_1 and λ_2 are superposed. The resulting composite spectrum is sampled at 10 nm interval over the 450 nm–700 nm range. Because the sampling step of 10 nm exceeds the laser's full width at half maximum (FWHM), the discrete spectrum takes on the appearance of a superposition of impulse-like peaks. It is important to note, however, that this impulse-like profile is a consequence of coarse spectral sampling and does not result from the use of actual impulse functions. This spectrum is then replicated spatially across a 128 × 128 grid to construct a hyperspectral data cube, which serves as the input signal for the resolution test.

This sparse signal is encoded by using a simulated F–P cavity array, constructed as a 128 × 128 random pattern modulated by the simulated cavity transmittance (Fig. S6a). The dual-peak separation simulations are systematically implemented at three central wavelength channels (λ_1 = 470 nm, 500 nm, and 600 nm). For each wavelength band, λ_2 is stepped through increasing separations with respect to λ_1, with reconstruction carried out at each step, until the two peaks become distinguishable in the reconstructed spectra.

The results of the resolution assessment are plotted in Fig. S21b–g. Fig. S21b–d displays representative cases in which the reconstructed dual peaks are just able to be resolved, i.e., they exhibit the trend to merge into a single continuous profile but with a distinguishable dip. In comparison, Fig. S21e–g shows the cases in which the peak pairs can be resolved with a distinguishable dip, where two distinct maxima are clearly identified. Therefore, when the wavelength separation between λ_1 and λ_2 is 60 nm, the dual peaks can be successfully resolved for all the three wavelength channels. As a result, the spectral resolution of the hyperspectral imaging system is ~ 60 nm across the visible wavelength range, which is larger than the spectral peak sensitivity (~ 2 nm) of the system. It might be attributed to the broad bandwidth of the F–P cavity color filters. Therefore, the hyperspectral imaging system based on the F–P cavity array is suitable to applied to areas where the spectrum of the object is broad and continuous.

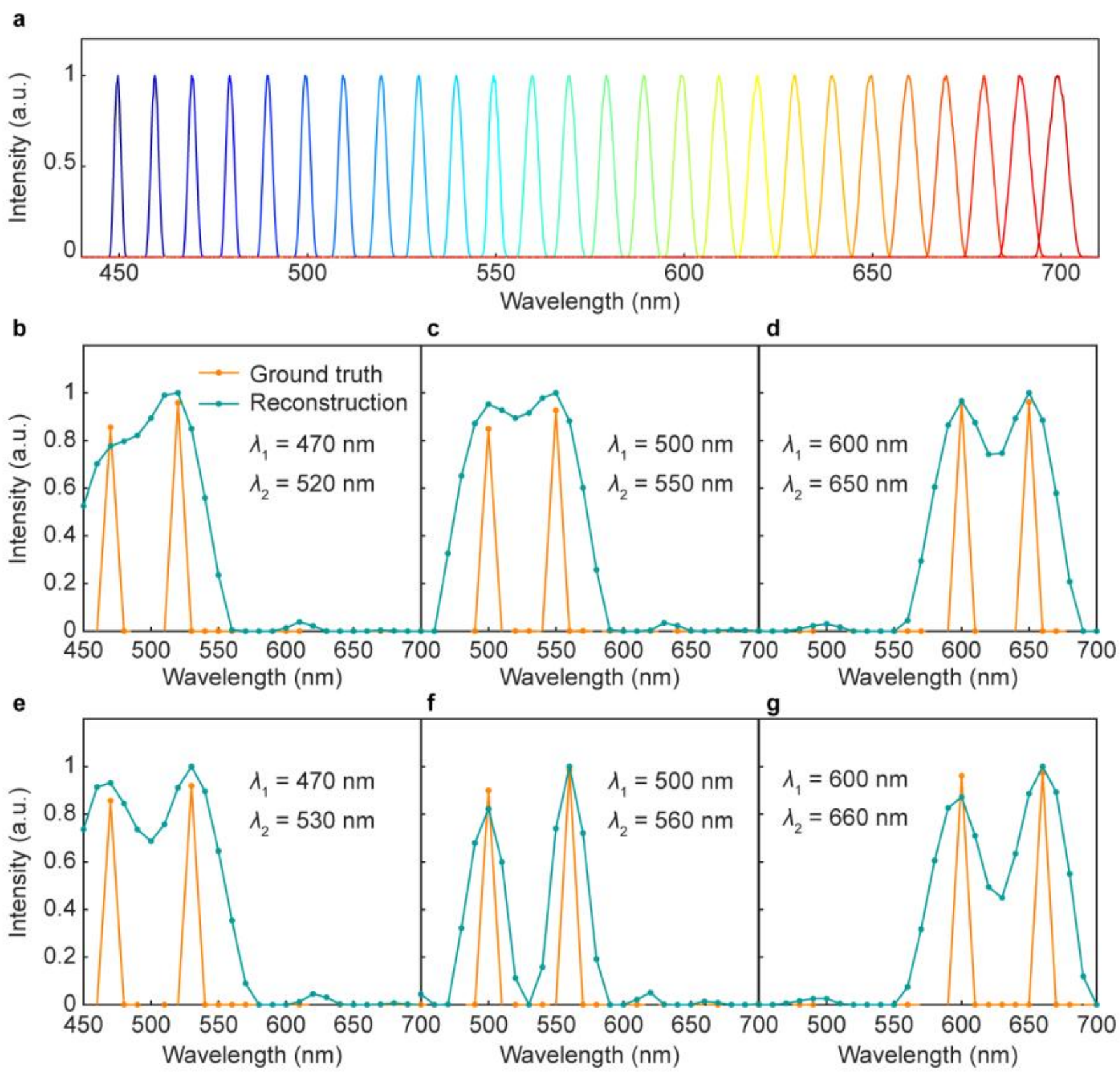

Figure S21. Assessment of spectral resolution via dual-peak separation test. (a) Normalized measured spectra of the supercontinuum laser. (b–d) Representative cases of the critical condition where the dual peaks are just able to be resolved. The wavelength separation is 50 nm, and the central wavelength of the first peak λ_1 is 470 nm, 500 nm, and 600 nm, respectively. (e–g) Representative cases where the dual peaks are resolved with a distinguishable dip when the wavelength separation is 60 nm.

To further explore the resolution limit of our system, we fitted the measured spectra of the supercontinuum laser with a peak spacing of 10 nm (Fig. S22a, upper panel) to generate a set of laser spectra with a peak spacing of 5 nm (Fig. S22a, lower panel). The generated spectra follow a Gaussian lineshape with a wavelength-dependent bandwidth. The relationship between the peak position and the FWHM of these laser spectra is plotted in Fig. S22b. Using the fitted spectra with a 5 nm spectral interval as the incident light, we perform the reconstruction calculation on a 128 × 128 grid to evaluate the system's capability to resolve dual-peak incident lasers. At the three central wavelength (λ_1 = 470 nm, 500 nm, and 600 nm), λ_2 is gradually increased, until the two peaks became distinguishable after reconstruction.

The main resolution assessment results are presented in Fig. S22c–h, where two spectrally adjacent peaks are synthesized and processed through the system. Panels in Fig. S22c–e display the representative cases in which the separation of the dual peaks approach the limit at the central wavelength of 470 nm, 500 nm, and 600 nm. In comparison, panels in Fig. S22f–h show the clearly resolved dual peaks at the three wavelength bands. It can be found that the minimum resolvable wavelength spacing of our system is ~ 60 nm at the wavelength of 470 nm, 500 nm, and 600 nm. Comparing the reconstruction results obtained with 10 nm and 5 nm spectral intervals of the supercontinuum laser, we find that the hyperspectral imaging system yields

similar dual-peak reconstruction capability in the two cases. This observation confirms that the system's spectral resolution limit for dual-peak resolving is approximately 60 nm. This limitation is fundamentally related to the intrinsic transmission bandwidth of each F–P cavity.

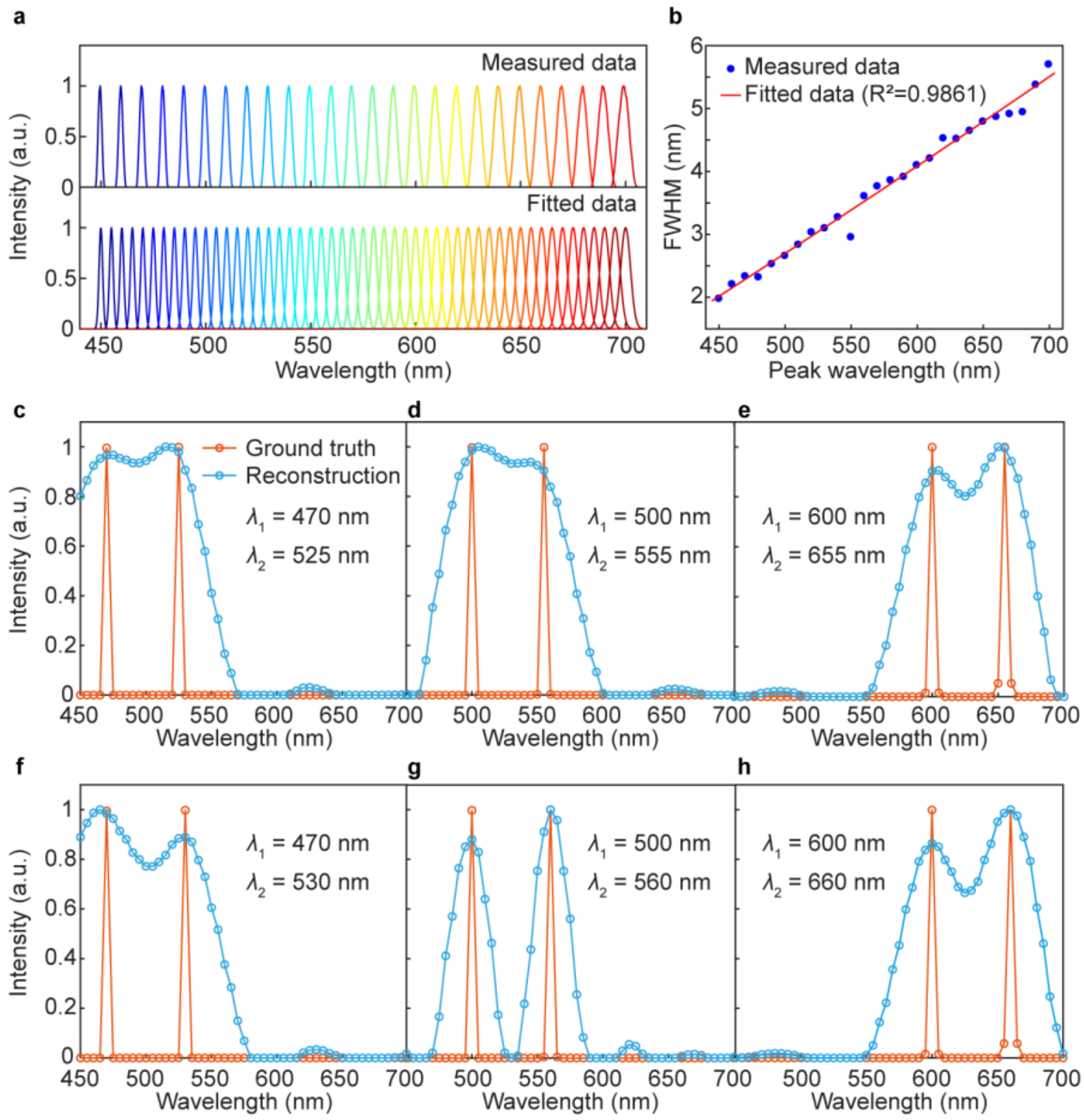

Figure S22. Assessment of the spectral resolution via dual-peak separation test. (a) Normalized measured and fitted spectra of the supercontinuum laser. (b) Peak wavelength versus full width at half maximum (FWHM) of the laser source. (c–e) Representative cases of the critical condition where the dual peaks are just resolved. The wavelength separation is ~ 55 nm, and the central wavelength of the first peak is 470 nm, 500 nm, and 600 nm, respectively. (f–h) Representative cases where the dual peaks are resolved with clear dips when the wavelength separation is 60 nm.

S15. Alignment process between the F–P color filter array and the CMOS sensor pixels

The alignment accuracy between the F–P cavity array and the sensor pixels directly affects the fidelity of the reconstructed spectra. Therefore, we assess the alignment quality in real time by monitoring the live signal from the CMOS sensor. A tungsten-halogen light source (SLS201L/M, Thorlabs) is first expanded and used to illuminate the F–P cavity array at normal incidence. The F–P cavity array device (Fig. S23a) is mounted on a three-axis translation stage (M-462-XYZ-M, Newport) combined with a rotation mount (PRM1/M, Thorlabs) to provide both translational and rotational degrees of freedom. A relay lens (#45-760, Edmund) is fixed in a V-clamp mount (C1513, Thorlabs). The CMOS sensor (CS165MU, Thorlabs) is placed on a three-axis translation stage (PT3/M, Thorlabs), and an additional rotation mount (KS1RS, Thorlabs) is incorporated to enable multi-axis adjustment of its position and orientation. By observing the real-time CMOS sensor output, we iteratively adjust the relative position of the F–P cavity array, the relay lens, and the CMOS sensor until the signal at the four edges of the sensor becomes sharp and symmetric. This condition indicates that pixel-level alignment between the F–P cavity array and the CMOS sensor has been achieved. As shown in Fig. S23b,c, when the alignment is finished, the edges of the CMOS sensor image becomes sharp, straight, and well-defined. After alignment, all components are mechanically locked in place, as shown in Fig. S24. The entire experimental setup is built on an optical table with excellent stability. After the optical alignment, the supercontinuum laser is delivered to the optical path for calibration by using a reflective mirror and a flip mirror (Fig. S25). The flip mirror is flipped down during the previous alignment process to let the light from the tungsten-halogen source pass through. After the calibration process, the color checker (X-rite color checker classic mini), the imaging lens (#59-870, Edmund), and the white light LED source (Effilux) are added to verify the hyperspectral imaging performance of the system (Fig. S26), while the position of the F–P cavity array, the relay lens, and the CMOS sensor remains unchanged.

After the hyperspectral imaging, no additional alignment algorithm is required in the reconstruction process. The hyperspectral imaging apparatus is constructed in the laboratory, and its stability highly relies on the mechanical performance of the optical table (M-RS2000-510-12, Newport). For practical applications, the F–P cavity array could be bonded to the CMOS sensor by using optical adhesive or monolithically fabricated on the CMOS sensor, which would offer better imaging stability.

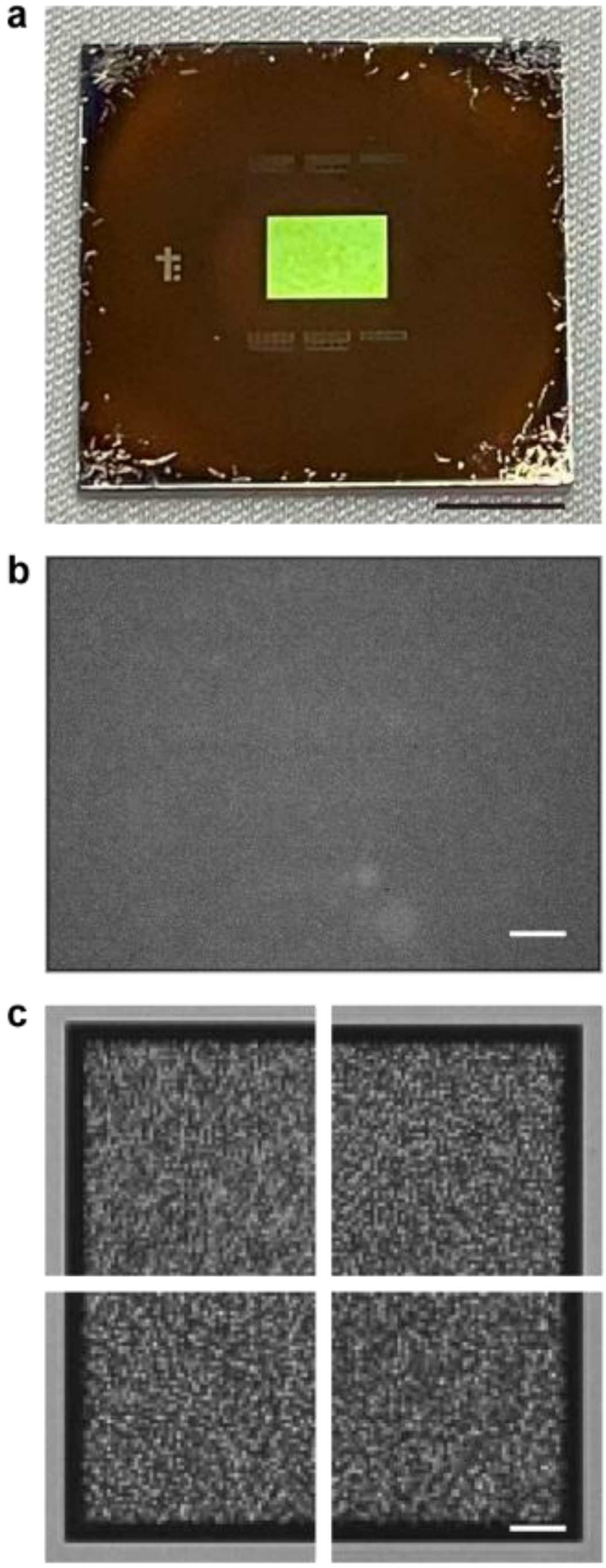

Figure S23. Photograph of the F–P cavity array and the sensor image during the alignment process. (a) Photograph of the F–P cavity array. The area size of the device is 4933.5 μm × 3691.5 μm, corresponding to 1430 × 1070 pixels. The pixel size of the F–P cavity array is 3.45 μm × 3.45 μm, which matches that of the CMOS sensor (CS165MU, Thorlabs). (b) The sensor image during the alignment process. A border is added around the active area of the F–P cavity array to assist observation during the optical alignment. (c) Four corners of panel (b). When the image of the black border becomes sharp and straight, the F–P cavity array device is aligned well with the CMOS sensor. Scale bar: 5 mm in (a), 500 μm in (b), and 50 μm in (c).

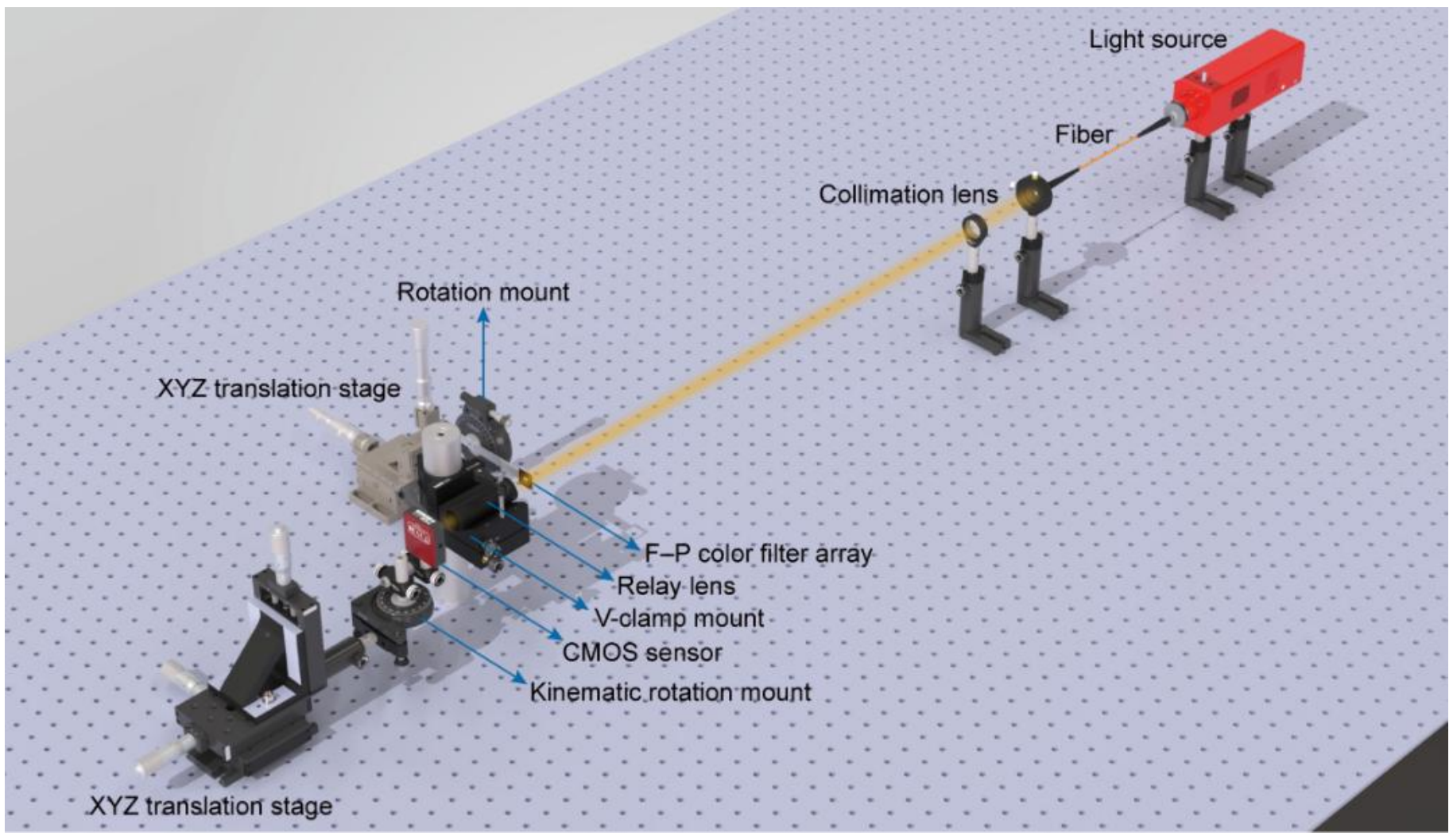

Figure S24. Schematic diagram of the experimental setup for the optical alignment. Optical table (M-RS2000-510-12); Stabilized tungsten-halogen light source (SLS201L/M, Thorlabs); Fiber patch cable (M153L01, Thorlabs); Fiber adapter (S1SMA, Thorlabs); Collimation lens (LA1131, *f* = 50 mm, Thorlabs); High-precision rotation mount (PRM1/M, Thorlabs); Relay lens (#45-760, Edmund); Post kinematic V-clamp mount (C1513, Thorlabs); XYZ translation stage (462-XYZ-M, Newport); CMOS sensor (CS165MU, Thorlabs); Kinematic rotation mount (KS1RS, Thorlabs), and XYZ translation stage (PT3/M, Thorlabs).

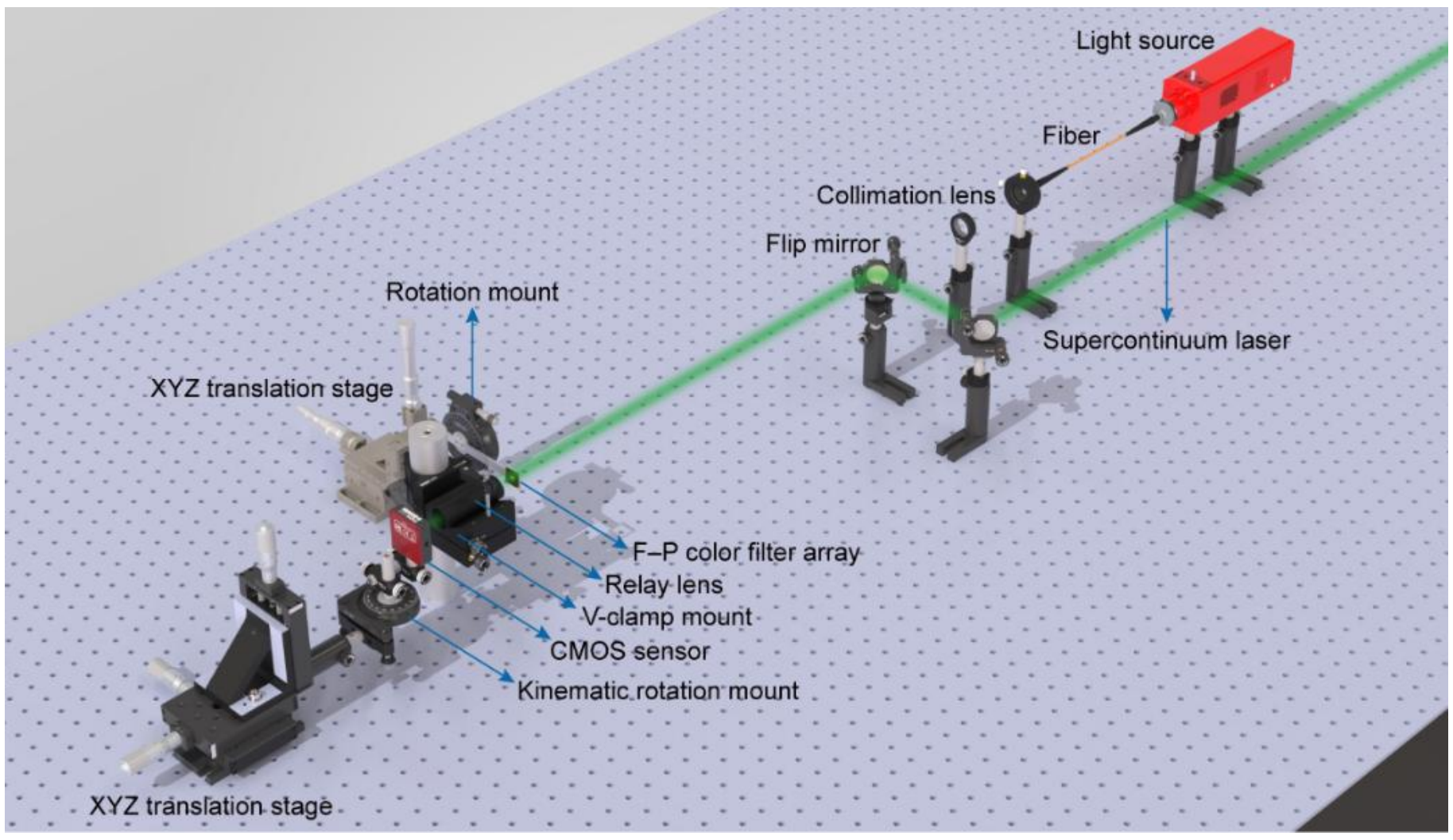

Figure S25. Schematic diagram of the experimental setup for calibrating the measurement matrix of the hyperspectral imaging system. After alignment, the supercontinuum laser is delivered to the calibration path by using a reflective mirror and a flip mirror. In this step, the flip mirror is flipped up to send the supercontinuum laser into the system. In the alignment process, the flip mirror is flipped down to allow the light from the tungsten-halogen light source to pass through.

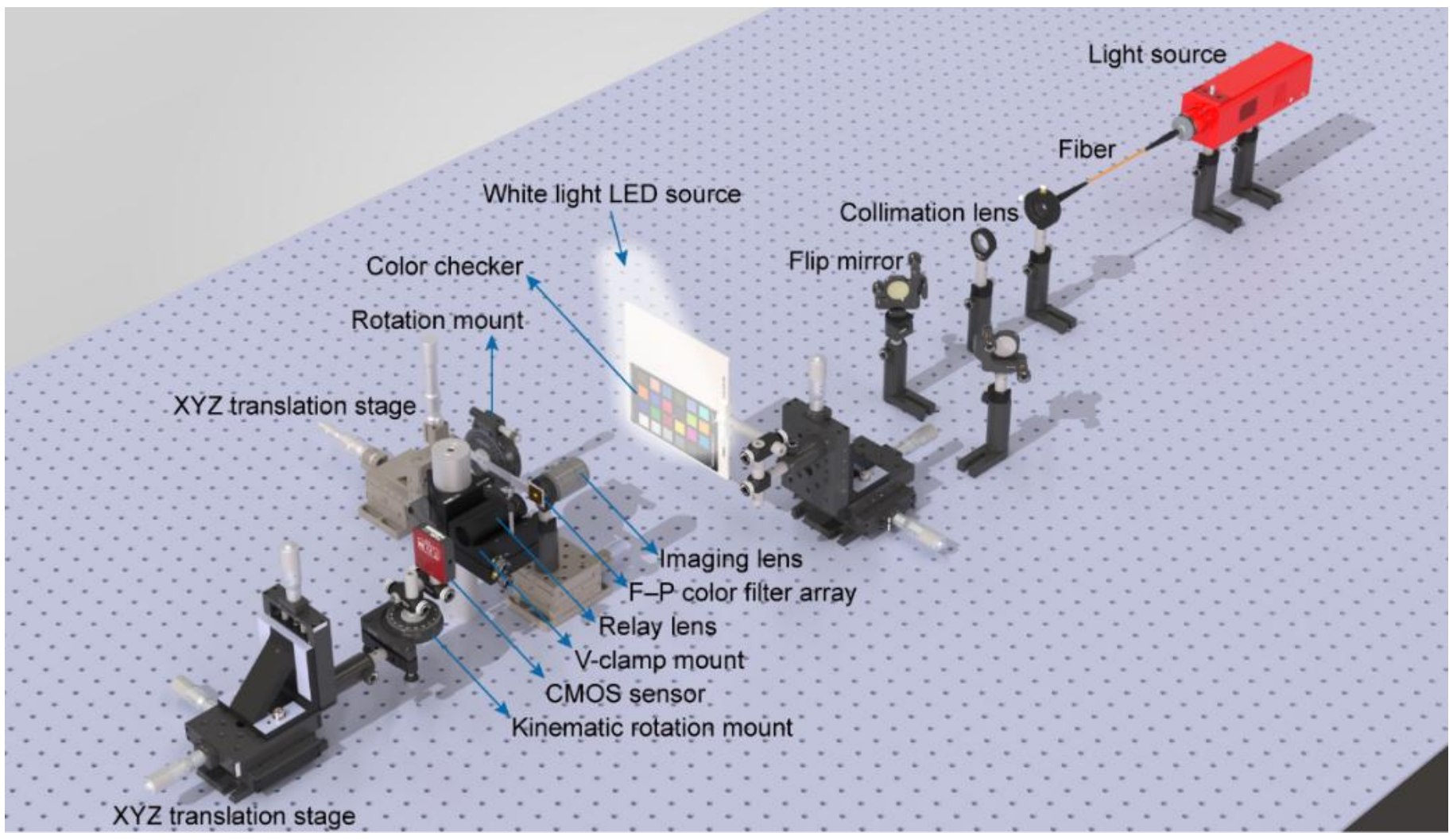

Figure S26. Schematic diagram of the experimental setup for the hyperspectral imaging of the color checker. The imaging lens (#59-870, Edmund), color checker (X-rite color checker classic mini), and the white light LED source (Effilux) are added into the system, while the position of the F–P cavity array, the relay lens, and the CMOS sensor remains unchanged.

References

1. Wang Z, Yi S, Chen A *et al*. Single-shot on-chip spectral sensors based on photonic crystal slabs. *Nat Commun* 2019; **10**: 1020.
2. Yako M, Yamaoka Y, Kiyohara T *et al*. Video-rate hyperspectral camera based on a CMOS-compatible random array of Fabry–Pérot filters. *Nat Photonics* 2023; **17**: 218–23.
3. Bioucas-Dias JM, Figueiredo MAT. A new TwIST: Two-step iterative shrinkage/thresholding algorithms for image restoration. *IEEE Trans Image Process* 2007; **16**: 2992–3004.
4. Rakić AD, Djurišić AB, Elazar JM *et al*. Optical properties of metallic films for vertical-cavity optoelectronic devices. *Appl Opt* 1998; **37**: 5271–83.
5. Columbia Imaging and Vision Laboratory. *Multispectral Image Database*. https://cave.cs.columbia.edu/repository/Multispectral (5 August 2026, date last accessed).
